# Association between sepsis and all-cause and cause-specific premature mortality: a prospective cohort study

**DOI:** 10.3389/fpubh.2025.1666675

**Published:** 2025-11-14

**Authors:** Wenhui Kang, Jiyong Zhong, Fei Wang, Wulin Li, Zhenfeng Dou, Shaoguan Huang, Shaohua Yin, Lei Yuan, Dali You

**Affiliations:** 1 Department of Emergency and Critical Care Medicine, Jiading District Central Hospital Affiliated Shanghai University of Medicine and Health Sciences, Shanghai, China; 2 Department of Medical Engineering, Peking University Third Hospital, Beijing, China; 3 Department of Health Management, Naval Medical University, Shanghai, China

**Keywords:** sepsis, premature mortality, association, cohort study, UK biobank

## Abstract

**Objective:**

This study aimed to examine the association between sepsis, including its subtypes, and all-cause and cause-specific premature mortality.

**Methods:**

This population-based prospective cohort study included 371,558 participants from the UK Biobank recruited between 2006 and 2010. Sepsis was identified from hospital records using ICD-10 codes. Cox proportional-hazards models estimated adjusted hazard ratios (aHRs) and 95% confidence intervals (CIs) for premature mortality.

**Results:**

Among 371,558 participants, 47,149 (12.7%) were diagnosed with sepsis, including 21,148 with implicit sepsis, 620 with explicit sepsis, and 25,381 with both. Sepsis was associated with a higher risk of all-cause premature mortality (aHR 2.36, 95% CI 2.26–2.46). Cause-specific analyses showed elevated risks for cardiovascular (aHR 2.35, 95% CI 2.18–2.54), respiratory (aHR 7.30, 95% CI 6.23–8.55), cancer-related (aHR 1.76, 95% CI 1.66–1.87), and infection-related premature mortality (aHR 9.75, 95% CI 6.97–13.62). Participants with explicit sepsis alone had elevated risk of all-cause mortality (aHR 1.72, 95% CI 1.21–2.45), which was lower than implicit sepsis alone (aHR 2.05, 95% CI 1.94–2.17) and highest for those with both implicit and explicit sepsis (aHR 2.60, 95% CI 2.48–2.73). Risks were more pronounced in participants with older age, multiple comorbidities, and unhealthy lifestyle (*P*_interaction_ < 0.001).

**Conclusion:**

Sepsis, especially implicit and combined implicit-explicit sepsis, was associated with increased risks of all-cause and cause-specific premature mortality. These associations were stronger in older participants, those with comorbidities, and unhealthy lifestyles.

## Introduction

Premature mortality, defined as death occurring before the age of 70, reflects a country’s health achievements, with substantial implications for both individuals and healthcare systems (1). Globally, premature mortality accounts for a considerable proportion of total deaths (2–4), with noncommunicable diseases such as cardiovascular disease, cancer, and respiratory diseases often considered as predominant causes (5). However, there is an increasing awareness that infectious diseases, particularly sepsis, may also play a critical role in driving early mortality rates (6). Despite advances in medical care, sepsis remains a major cause of morbidity and mortality worldwide, contributing to an estimated 11 million sepsis-related deaths in 2017, which accounted for 20% of all global deaths for that year (7).

Sepsis, a life-threatening condition triggered by a dysregulated host response to infection, presents a significant clinical challenge due to its complex pathophysiology and the need for rapid diagnosis and intervention (8). In the United Kingdom, sepsis is a leading cause of death, contributing to approximately 48,000 deaths annually (9). It is an acute, life-threatening condition that can cause multi-organ injury, impair multiple systems, and accelerate chronic disease progression, resulting in both short-term and long-term health consequences (10, 11). The risk of death is particularly high in the weeks and months following a sepsis diagnosis, highlighting the importance of understanding both the short-term and long-term impacts of sepsis on survival (12, 13).

While the short-term outcomes of sepsis, including its association with higher in-hospital mortality and post-discharge complications, are well-documented, the long-term impacts remain less understood. A meta-analysis of global data estimated the in-hospital mortality rate for sepsis at approximately 26.7% (14), with higher rates reported in low- and middle-income countries (15). Additionally, a population-based cohort study of 144,503 sepsis survivors showed a 1.7 to 2.9-fold increased risk of death within 1 year post-discharge (16). However, there is a relative paucity of study exploring the long-term effects of sepsis on premature mortality, leaving a significant gap in understanding sepsis’s contribution to all-cause and cause-specific premature mortality over extended periods. Collecting reliable population-level data presents challenges (17), and existing studies often fail to account for potential confounding factors, such as pre-existing comorbidities and socioeconomic status, limiting the generalizability of findings.

This study aimed to investigate the association between sepsis and both all-cause and cause-specific premature mortality in a large, prospective cohort from the UK Biobank. Using comprehensive health data and long-term follow-up, this study might provide a new understanding of how sepsis influences premature mortality risk and identify potential opportunities for targeted interventions that could improve long-term outcomes for sepsis survivors.

## Materials and methods

### Study design and participants

This nationwide, population-based cohort study used data from the UK Biobank (UKB, application 99,709), a dataset established to support a wide range of studies aimed at improving the prevention, diagnosis, and treatment of various diseases, as well as to examine the long-term effects of different exposures on health outcomes (18). The cohort consisted of participants recruited from 22 dedicated assessment centers across England, Scotland, and Wales between 2006 and 2010, encompassing approximately half a million individuals aged 39 to 71 years at the time of recruitment. All participants provided informed consent for their data to be used in future research. The UK Biobank received ethical approval from the UK North West Multi-Center Research Ethics Committee (11/NW/0382), and the study was conducted in accordance with the principles of the Declaration of Helsinki.

For this study, participants with available data on both sepsis diagnosis and death records were included. Individuals with missing data on key covariates, including sociodemographic factors, health status, lifestyle factors were excluded. Participants were followed from the date of recruitment (baseline UK Biobank assessment, 2006–2010) until death or the end of the follow-up period, whichever occurred first.

### Sepsis measurement

Participants diagnosed with sepsis were identified using International Classification of Diseases, Tenth Revisions, Clinical Modification (ICD-10-CM) codes obtained from primary and secondary diagnosis in hospitalization records (7). Individuals who met the sepsis criteria during any hospitalization were classified as septic, while those who did not met these criteria were classified as non-septic. For participants with multiple hospitalizations, the first hospitalization with a diagnosis of sepsis after baseline was considered as the index hospitalization, and hospitalizations prior to UK Biobank recruitment, including those occurring in childhood, were not included. If sepsis was not diagnosed, the first hospitalization was still considered as the index event for classification.

Following previous epidemiological studies using administrative data (19–21), sepsis was further categorized as explicit sepsis, implicit sepsis, or both explicit and implicit sepsis based on ICD-10 diagnosis codes (22) (Table 1). Explicit sepsis was defined by diagnosis codes specific to sepsis or septic shock (Supplementary Table S1). For example, the ICD-10 code O03.300×001, indicating “Spontaneous abortion, Incomplete, with septic shock,” was classified as explicit sepsis. Implicit sepsis was identified using a validated algorithm that required coexistence of at least one diagnosis of infection (Supplementary Table S2) and one or more diagnoses of acute organ dysfunction during the hospitalization (Supplementary Table S3) (7, 21). This approach was validated through a prospective study assessing the diagnostic accuracy of the quick Sequential Organ Failure Assessment score for sepsis in both general wards and Intensive Care Units (23).

**Table 1:** Summary of ICD-10-based classification of sepsis.

Classification	Definition	ICD-10 codes
Explicit sepsis	Hospitalizations with a direct diagnosis code for sepsis or septic shock	A01–A03, A09, A20–A28, A32, A38–A42, A48, A54, A93, A98, B00, B37, B49, F05–F06, J15, J18, J95, K85, O03–O04, O08, O75, O85, O88, O98, P36–P37, R57, R65, T80–T81, T88
Implicit sepsis	Co-occurrence of ≥1 infection code and ≥1 acute organ dysfunction code during the same hospitalization	Infection code: 00–A09, A15–A28, A30–A32, A35–A43, A46, A48–A54, A65–A67, A69, A75, A77–A96, A98–A99, B33–B36, B38–B60, B64–B69, B70–B83, B85, B95–B96, G00–G03, G05–G06, G08, I30, I32–I33, I39, I41, I52, I60, I70–I80, J01–J06, J13–J15, J17–J18, K23, K35–K37, K57, K61, K63, K65, K67, K75, K77, K81, K90, N10–N12, N15, N29, N30, N33–N34, N39, N41, N51, N72, N74, L03, L04, L08, L98–L99, M00–M01, M86, M89–M90, T80–T85; acute organ dysfunction code: A01–A03, A09, A20–A22, A24, A26–A28, A32, A38–A42, A48–A49, A54, A88, A93, A98, B00 B15–B17, B19, B25, B37, B49, D61, D65, D68–D69, D71, D76, E80, E86–E87, F05–F06, G93, I51, I95, I99, J15, J18, J80–J81, J95–J96, J98, K71–K72, K76, K91, N17, N19, O03–O04, O08, O85, O88, P22, P28–P29, P36–P37, R09, R34, R39–R40, R41, R45, R55, R57, R65, R68, R94, T80–T81, T88, U04
Both explicit and implicit sepsis	Meeting both criteria during the index hospitalization	Combination of codes satisfying both categories

### Premature mortality measurement

Premature mortality was defined as death occurring before the age of 70 years, consistent with definitions used in previous studies (24, 25). Participants who died at or after the age of 70, or who were event-free at the end of the study, were considered censored cases in the analysis. Cause-specific mortality was determined according to the underlying cause of death as recorded on death certificates, which were coded using ICD-10 codes. The causes of death were categorized into the following groups: cardiovascular disease (I00-I99), cancer (C00-D48), respiratory disease (J00-J99), infection (A00-B99), and other causes, including external causes such as accidents or injuries (26, 27). Deaths from other causes were treated as censoring events.

### Covariates measurement

Covariates included sociodemographic factors, lifestyle factors, and health status, with data obtained from the UKB baseline assessment and linked health records. Gender was self-reported at baseline and categorized as either female or male. Age at recruitment was calculated by subtracting the participant’s date of birth from the date of recruitment and categorized into four groups: ≤54 years, 55–59 years, 60–64 years, and ≥65 years. Ethnicity was self-reported and classified as either White or Non-white. Education was assessed based on the highest qualification reported by participants and was categorized as having a college or university degree or having other educational levels (including secondary education and below). The Townsend deprivation index, a composite measure of socioeconomic status, was calculated based on participants’ postal codes and categorized into quintiles ranging from 1 (least deprived) to 5 (most deprived). Body mass index (BMI) at recruitment was calculated from height (measured in meters) and weight (measured in kilograms) measurements taken at baseline. Based on the World Health Organization guidelines, participants were classified into four BMI categories: <18.5 (underweight), 18.5–24.9 (normal weight), 25–29.9 (overweight), and ≥30 (obese). A composite lifestyle score was derived from participants’ responses to questions on smoking status, alcohol consumption, physical activity, television viewing time, frequency of food consumption, and sleep patterns. Each lifestyle factor was assigned a score, with higher total scores indicating a less healthy lifestyle. Participants were categorized into three groups based on their lifestyle score: most healthy (0–2), moderately healthy (3–5), and least healthy (6–9) (Supplementary method S1; Supplementary Table S4). The presence of cardiovascular disease at baseline was determined based on self-reported health status (e.g., angina, stroke, heart disease, hypertension), categorized as no or yes. The Charlson comorbidity index (CCI), a widely used method for categorizing comorbidities based on the ICD-10 diagnosis codes, was calculated and categorized into three groups: 0 (no comorbidities), 1 (one comorbidity), and ≥2 (two or more comorbidities; Supplementary Table S5). This index was used to account for the burden of chronic disease and its potential impact on mortality risk (28).

### Statistical analysis

Descriptive statistics were used to summarize the baseline characteristics of the study participants. Categorical variables were reported as frequencies and percentages, and continuous variables were presented as means with standard deviations (SDs) or medians with interquartile ranges (IQRs) as appropriate. The incidence rates of all-cause and cause-specific premature mortality were calculated as the number of deaths per 1,000 person-years of follow-up.

Cox proportional hazards regression models were conducted to estimate hazard ratios (HRs) and 95% confidence intervals (CIs) for the association between sepsis and all-cause and cause-specific premature mortality. The models were adjusted for potential confounders in a stepwise manner. The Directed Acyclic Graph (DAG) was plotted to identify potential confounders, informing the selection of variables for adjustment in multivariable models (Supplementary Figure S1). Model 1 was adjusted for sociodemographic variables, including age, gender, ethnicity, education, Townsend deprivation index, BMI, smoke status, and alcohol drinking; Model 2 included additional adjustments for health status, including cardiovascular disease and diabetes mellitus at recruitment, as well as comorbidities indicated by the CCI; and the final model (Model 3) further adjusted for lifestyle score. The proportional hazards assumption was assessed using Schoenfeld residuals, with no significant violations detected for any covariate (all *p* > 0.05, Supplementary Table S6).

To ensure the robustness of the findings, sensitivity analyses were conducted, including (1) excluding participants with a history of cancer at baseline to mitigate potential reverse causation, (2) redefining premature mortality as death occurring before age 75 years, (3) using propensity score overlap weighting, with all relevant variables shown in Supplementary Table S7; post-weighting standardized mean differences were less than 0.001 (29), using multiple imputation approach to hand missing data, and using Fine–Gray subdistribution hazard models treating deaths from other causes as competing events.

To explore potential effect modification, stratified analyses were performed by age, CCI, and lifestyle score. Interaction effects were tested by adding an interaction term between sepsis and each stratified variable into the multivariable models.

All statistical analyses were conducted using SAS version 9.4 (SAS Institute Inc.), and a two-sided *p*-value of less than 0.05 was considered statistically significant.

## Results

**Figure 1:**
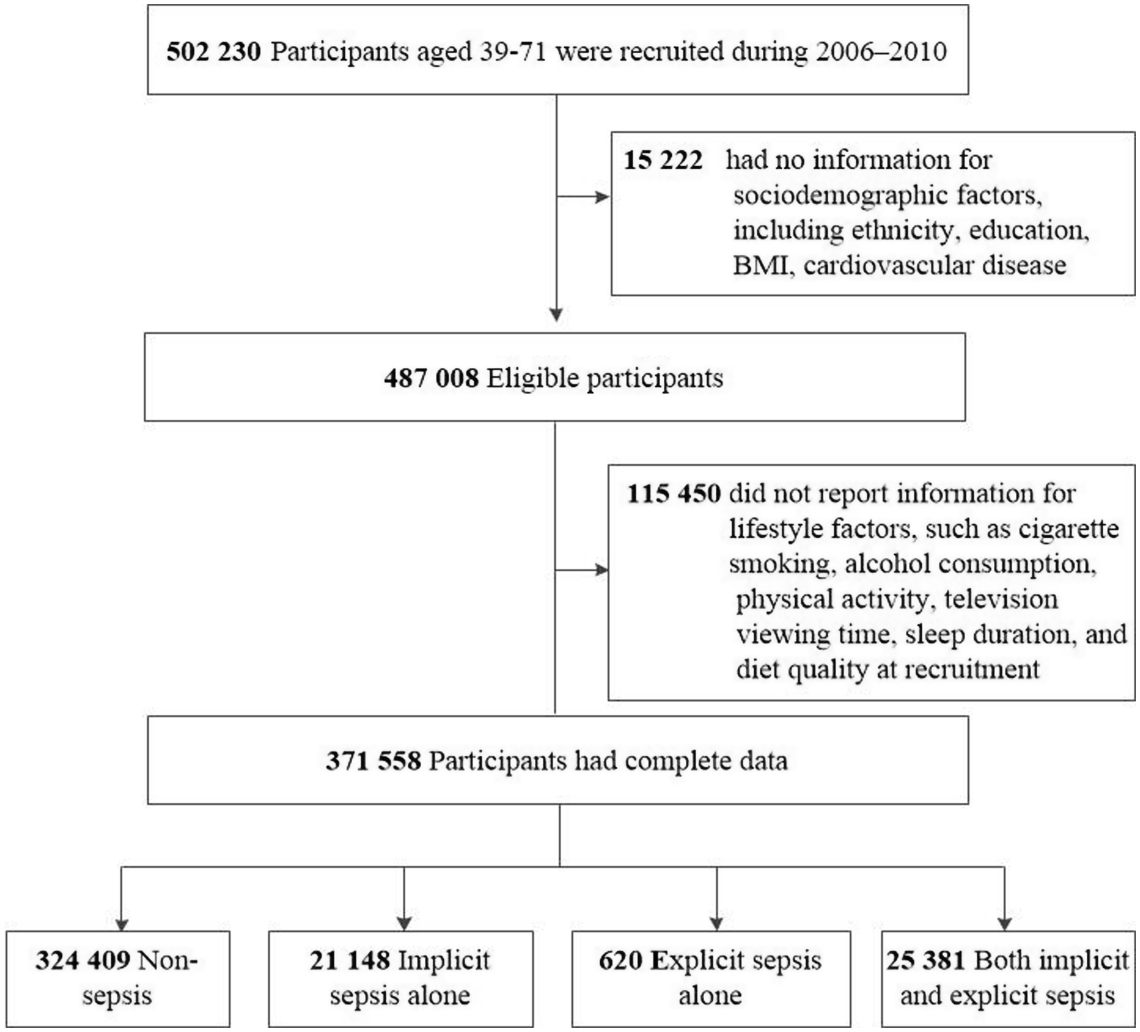
Flowchart of study participants.

Among the UKB cohort of 502,230 participants, 130,672 (26.0%) individuals were excluded due to missing data for ethnicity (*n* = 2,775), education (*n* = 9,019), BMI (*n* = 2,508), cardiovascular disease (*n* = 920), and lifestyle factors (*n* = 115,450), resulting in a final study sample of 371,558 participants (Figure 1). During a median follow-up of 15.0 years (IQR 14.0–16.0), totaling 5,379,547 person-years, there were 10,479 premature deaths by December 29, 2022, accounting for 35.0% of all deaths recorded (Table 2).

**Table 2:** Baseline characteristics of participants with and without sepsis.

Characteristic	No sepsis	Any sepsis	Total	*p* value
Participants	324,409	47,149	371,558	
Sociodemographic				
Gender				<0.001
Female	173,210 (53.4)	21,119 (44.8)	194,329 (52.3)	
Male	151,199 (46.6)	26,030 (55.2)	177,229 (47.7)	
Age at recruitment (years)	55.6 ± 8.1	60.0 ± 7.3	56.2 ± 8.1	<0.001
Age group				<0.001
≤54	139,444 (43)	10,259 (21.8)	149,703 (40.3)	
55–59	59,956 (18.5)	7,452 (15.8)	67,408 (18.1)	
60–64	74,585 (23.0)	13,749 (29.2)	88,334 (23.8)	
≥65	50,424 (15.5)	15,689 (33.3)	66,113 (17.8)	
Ethnicity				<0.001
White	309,553 (95.4)	45,231 (95.9)	354,784 (95.5)	
Non-white	14,856 (4.6)	1918 (4.1)	16,774 (4.5)	
Education				<0.001
College or University degree	42,495 (13.1)	4,976 (10.6)	47,471 (12.8)	
Others	281,914 (86.9)	42,173 (89.4)	324,087 (87.2)	
Townsend deprivation index quintile				0.447
1 (least deprived)	66,390 (20.5)	8,174 (17.3)	74,564 (20.1)	
2	65,208 (20.1)	8,667 (18.4)	73,875 (19.9)	
3	65,435 (20.2)	9,009 (19.1)	74,444 (20.0)	
4	64,928 (20.0)	9,444 (20.0)	74,372 (20.0)	
5 (most deprived)	62,448 (19.2)	11,855 (25.1)	74,303 (20.0)	
BMI at recruitment (kg/m^2^)				<0.001
<18.5	1,564 (0.5)	318 (0.7)	1882 (0.5)	
18.5–24.9	112,694 (34.7)	12,126 (25.7)	124,820 (33.6)	
25–29.9	139,610 (43.0)	19,715 (41.8)	159,325 (42.9)	
≥30	70,541 (21.7)	14,990 (31.8)	85,531 (23)	
Lifestyle				
Lifestyle score category				<0.001
Most healthy	197,041 (60.7)	24,274 (51.5)	221,315 (59.6)	
Moderately healthy	120,862 (37.3)	21,044 (44.6)	141,906 (38.2)	
Least healthy	6,506 (2.0)	1831 (3.9)	8,337 (2.2)	
Smoking status				<0.001
Never	183,614 (56.6)	20,846 (44.2)	204,460 (55)	
Previous	110,802 (34.2)	19,522 (41.4)	130,324 (35.1)	
Current	29,993 (9.2)	6,781 (14.4)	36,774 (9.9)	
Alcohol drinking				<0.001
Never	11,363 (3.5)	2,285 (4.8)	13,648 (3.7)	
Previous	9,527 (2.9)	2,699 (5.7)	12,226 (3.3)	
Current	303,519 (93.6)	42,165 (89.4)	345,684 (93)	
Alcohol intake, times/week	1.5 (0.5–3.5)	1.5 (0.5–3.5)	1.5 (0.5–3.5)	<0.001
Television viewing time, h/day	2.0 (2.0–4.0)	3.0 (2.0–4.0)	3.0 (2.0–4.0)	<0.001
Sleep duration, h/day	7.0 (7.0–8.0)	7.0 (6.0–8.0)	7.0 (7.0–8.0)	<0.001
Fruit and vegetables intake, g/day	720 (480–960)	720 (480–960)	720 (480–960)	0.001
Oily fish intake, portions/week	1.0 (0.5–1.0)	1.0 (0.5–1.0)	1.0 (0.5–1.0)	<0.001
Red meat intake, portions/week	1.5 (1.5–2.5)	2.0 (1.5–3.0)	2.0 (1.5–2.5)	<0.001
Processed meat intake, portions/week	1.0 (0.5–3.0)	1.0 (0.5–3.0)	1.0 (0.5–3.0)	<0.001
Physical activity at moderate intensity				<0.001
No	179,044 (55.2)	24,340 (51.6)	203,384 (54.7)	
Yes	145,365 (44.8)	22,809 (48.4)	168,174 (45.3)	
Health status				
Cardiovascular disease at recruitment				<0.001
No	239,879 (73.9)	26,169 (55.5)	266,048 (71.6)	
Yes	84,530 (26.1)	20,980 (44.5)	105,510 (28.4)	
Charlson comorbidity index				<0.001
0	215,638 (66.5)	7,973 (16.9)	223,611 (60.2)	
1	78,913 (24.3)	15,123 (32.1)	94,036 (25.3)	
≥2	29,858 (9.2)	24,053 (51.0)	53,911 (14.5)	

Data are presented as number (percentage), mean (SD), or median (P25, P75).

The average age of the study participants was 56.2 (SD 8.1) years, with the majority being female (52.3%) and White (95.4%), and most having an education level below a college or university degree (87.2%). Among the cohort, 47,149 (12.7%) individuals were diagnosed with sepsis, including 21,148 with implicit sepsis alone, 620 with explicit sepsis alone, and 25,381 with both. Baseline characteristics significantly differed between participants with sepsis and those without (Table 2). Individuals with sepsis were generally older, males, had a greater prevalence of overweight or obesity, more comorbidities, a higher incidence of pre-existing cardiovascular disease, and were more likely to have of unhealthy lifestyle behaviors (e.g., current smoking, longer television viewing time, shorter sleep duration, and higher red meat intake). A comparative analysis of characteristics among participants with sepsis showed differences between those with implicit, explicit, or both types of sepsis (Table 3). Generally, individuals with explicit sepsis had the lowest risk, while those with both implicit and explicit sepsis had the highest risk.

**Table 3:** Baseline characteristics of participants with sepsis according to the type of sepsis.

Characteristic	Explicit alone	Implicit alone	Both implicit and explicit	Total	*p* value
Participants	620	21,148	25,381	47,149	
Sociodemographic					
Gender					<0.001
Female	300 (48.4)	9,886 (46.7)	10,933 (43.1)	21,119 (44.8)	
Male	320 (51.6)	11,262 (53.3)	14,448 (56.9)	26,030 (55.2)	
Age at recruitment (years)	60.2 ± 8.3	60.0 ± 7.3	60.0 ± 7.3	60.0 ± 7.3	<0.001
Age group					<0.001
≤54	136 (21.9)	4,596 (21.7)	5,527 (21.8)	10,259 (21.8)	
55–59	64 (10.3)	3,360 (15.9)	4,028 (15.9)	7,452 (15.8)	
60–64	178 (28.7)	6,130 (29.0)	7,441 (29.3)	13,749 (29.2)	
≥65	242 (39.0)	7,062 (33.4)	8,385 (33.0)	15,689 (33.3)	
Ethnicity					<0.001
White	602 (97.1)	20,245 (95.7)	24,384 (96.1)	45,231 (95.9)	
Non-white	18 (2.9)	903 (4.3)	997 (3.9)	1918 (4.1)	
Education					<0.001
College or University degree	75 (12.1)	2,194 (10.4)	2,707 (10.7)	4,976 (10.6)	
Others	545 (87.9)	18,954 (89.6)	22,674 (89.3)	42,173 (89.4)	
Townsend deprivation index quintile					<0.001
1 (least deprived)	115 (18.5)	3,658 (17.3)	4,401 (17.3)	8,174 (17.3)	
2	117 (18.9)	3,905 (18.5)	4,645 (18.3)	8,667 (18.4)	
3	110 (17.7)	4,016 (19.0)	4,883 (19.2)	9,009 (19.1)	
4	134 (21.6)	4,180 (19.8)	5,130 (20.2)	9,444 (20.0)	
5 (most deprived)	144 (23.2)	5,389 (25.5)	6,322 (24.9)	11,855 (25.1)	
BMI at recruitment (kg/m^2^)					<0.001
<18.5	3 (0.5)	155 (0.7)	160 (0.6)	318 (0.7)	
18.5–24.9	208 (33.5)	5,574 (26.4)	6,344 (25.0)	12,126 (25.7)	
25–29.9	288 (46.5)	8,859 (41.9)	10,568 (41.6)	19,715 (41.8)	
≥30	121 (19.5)	6,560 (31.0)	8,309 (32.7)	14,990 (31.8)	
Lifestyle					
Lifestyle score category					<0.001
Most healthy	352 (56.8)	11,050 (52.3)	12,872 (50.7)	24,274 (51.5)	
Moderately healthy	256 (41.3)	9,283 (43.9)	11,505 (45.3)	21,044 (44.6)	
Least healthy	12 (1.9)	815 (3.9)	1,004 (4.0)	1831 (3.9)	
Smoking status					<0.001
Never	309 (49.8)	9,679 (45.8)	10,858 (42.8)	20,846 (44.2)	
Previous	244 (39.4)	8,528 (40.3)	10,750 (42.4)	19,522 (41.4)	
Current	67 (10.8)	2,941 (13.9)	3,773 (14.9)	6,781 (14.4)	
Alcohol drinking					<0.001
Never	26 (4.2)	1,097 (5.2)	1,162 (4.6)	2,285 (4.8)	
Previous	33 (5.3)	1,242 (5.9)	1,424 (5.6)	2,699 (5.7)	
Current	561 (90.5)	18,809 (88.9)	22,795 (89.8)	42,165 (89.4)	
Alcohol intake, times/week	1.5 (0.5–7.0)	1.5 (0.5–3.5)	1.5 (0.5–3.5)	1.5 (0.5–3.5)	<0.001
Television viewing time, h/day	3.0 (2.0–4.0)	3.0 (2.0–4.0)	3.0 (2.0–4.0)	3.0 (2.0–4.0)	<0.001
Sleep duration, h/day	7.0 (7.0–8.0)	7.0 (6.0–8.0)	7.0 (6.0–8.0)	7.0 (6.0–8.0)	<0.001
Fruit and vegetables intake, g/day	720 (480–960)	720 (480–960)	720 (480–960)	720 (480–960)	<0.001
Oily fish intake, portions/week	1.0 (0.5–3.0)	1.0 (0.5–1.0)	1.0 (0.5–1.0)	1.0 (0.5–1.0)	<0.001
Red meat intake, portions/week	2.0 (1.5–2.5)	2.0 (1.5–2.5)	2.0 (1.5–3.0)	2.0 (1.5–3.0)	<0.001
Processed meat intake, portions/week	1.0 (0.5–3.0)	1.0 (0.5–3.0)	1.0 (0.5–3.0)	1.0 (0.5–3.0)	<0.001
Physical activity at moderate intensity					<0.001
No	356 (57.4)	11,006 (52.0)	12,978 (51.1)	24,340 (51.6)	
Yes	264 (42.6)	10,142 (48.0)	12,403 (48.9)	22,809 (48.4)	
Health status					
Cardiovascular disease at recruitment					<0.001
No	376 (60.6)	11,598 (54.8)	14,195 (55.9)	26,169 (55.5)	
Yes	244 (39.4)	9,550 (45.2)	11,186 (44.1)	20,980 (44.5)	
Cancer at recruitment					
No	469 (75.6)	13,807 (65.3)	13,937 (54.9)	28,213 (59.8)	
Yes	151 (24.4)	7,341 (34.7)	11,444 (45.1)	18,936 (40.2)	
Charlson comorbidity index					<0.001
0	180 (29.0)	3,909 (18.5)	3,884 (15.3)	7,973 (16.9)	
1	228 (36.8)	7,186 (34.0)	7,709 (30.4)	15,123 (32.1)	
≥2	212 (34.2)	10,053 (47.5)	13,788 (54.3)	24,053 (51.0)	

Data are presented as number (percentage), mean (SD), or median (P25, P75).

**Figure 2:**
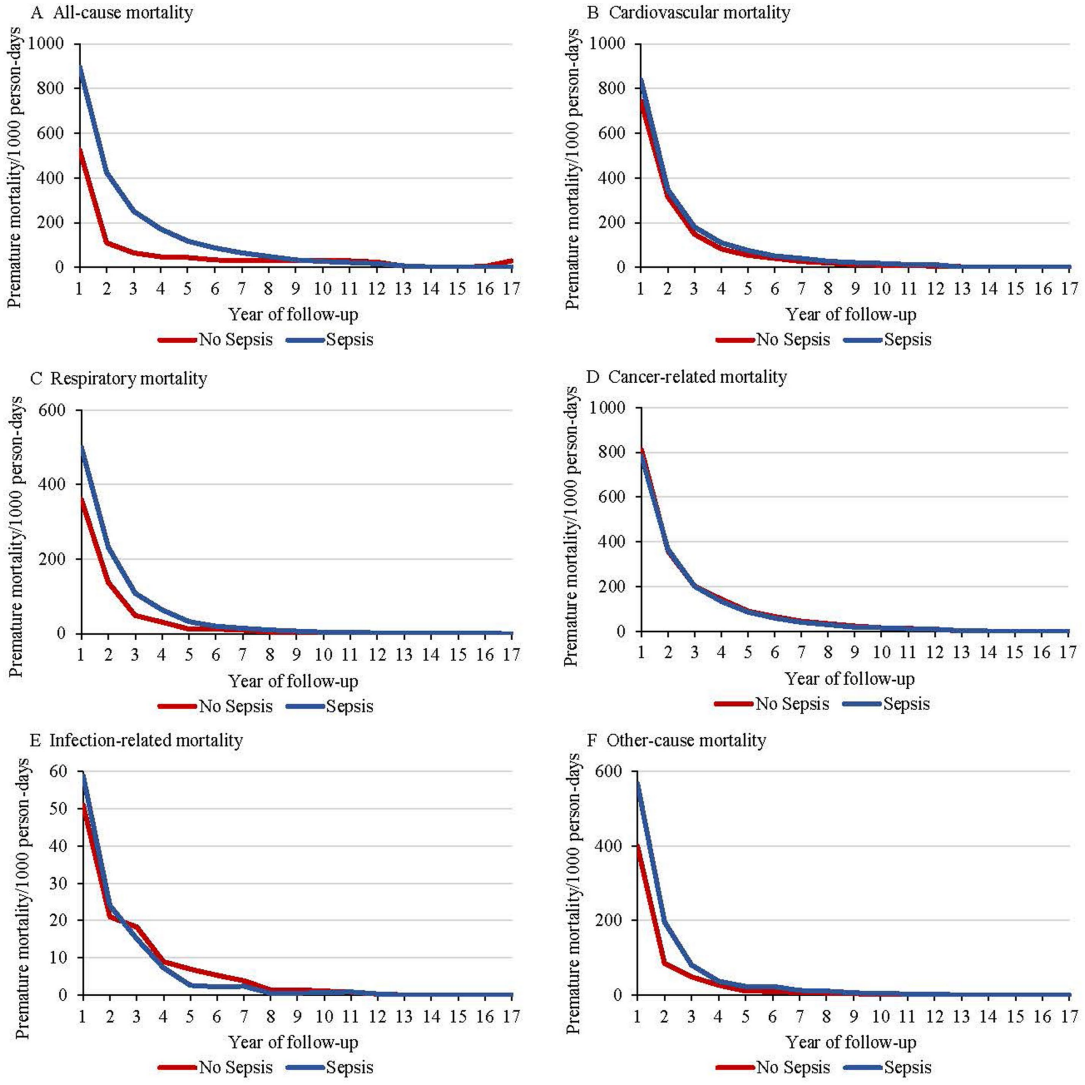
Premature mortality rates beginning with diagnosis among individuals with sepsis and without sepsis, including all-cause mortality **(A)**, cardiovascular mortality **(B)**, respiratory mortality **(C)**, cancer-related mortality **(D)**, infection-related mortality **(E)**, and other-cause mortality **(F)**.

Participants diagnosed with sepsis showed a markedly higher rate of all-cause premature mortality compared to those without sepsis, with similar patterns observed across specific causes of death, including cardiovascular disease, respiratory disease, infections, and other causes (Figure 2). The annual all-cause premature mortality rate for individuals with sepsis was 895 per 1,000 person-years in the first year following diagnosis, decreasing significantly to 435 per 1,000 person-years by the fifth year, and then stabilized or slightly declined. Most changes in cause-specific premature mortality occurred within the first 5 years.

In multivariable regression analyses adjusted for socioeconomic factors, sepsis was associated with a substantially higher risk of all-cause premature mortality (adjusted hazard ratio [aHR] 7.33, 95% confidence interval [CI] 7.05–7.64) compared to non-sepsis (Table 4). Although further adjustments for health status attenuated this association, it remained strong (aHR 2.37, 95% CI 2.27–2.47). Additional adjustments for lifestyle score made little difference to the association (aHR 2.36, 95% CI 2.26–2.46; Table 4). Sepsis was also associated with increased risks of cardiovascular (aHR 4.93, 95% CI 4.60–5.28), respiratory (aHR 16.35, 95% CI 14.16–18.86), cancer-related (aHR 8.55, 95% CI 8.06–9.07), infection-related (aHR 28.25, 95% CI 20.84–38.30), and other-cause (aHR 4.77, 95% CI 4.11–5.53) premature mortality, with risks persisting even after further adjustments for health status and lifestyle score (Table 4). Similar results were observed when premature mortality was alternatively defined as death before age 75 (Table 5).

**Table 4:** Event rates and adjusted hazard ratio for all-cause and cause-specific premature mortality, comparing any sepsis to participants without sepsis.

Model	No sepsis (ref)	Any sepsis
All-cause mortality		
Events/person-years	5487/4754324	4992/625223
Model 1—Sepsis (adjusted for sociodemographic factors)	1.00 (Ref)	7.33 (7.05–7.64)
Model 2 (adjusted for sociodemographic factors and health status)	1.00 (Ref)	2.37 (2.27–2.47)
Model 3 (adjusted for sociodemographic factors, health status, and lifestyle score)	1.00 (Ref)	2.36 (2.26–2.46)
Cardiovascular mortality		
Events/person-years	2138/4731578	1489/597372
Model 1—Sepsis (adjusted for sociodemographic factors)	1.00 (Ref)	4.93 (4.60–5.28)
Model 2 (adjusted for sociodemographic factors and health status)	1.00 (Ref)	2.38 (2.20–2.57)
Model 3 (adjusted for sociodemographic factors, health status, and lifestyle score)	1.00 (Ref)	2.35 (2.18–2.54)
Respiratory mortality		
Events/person-years	299/4718102	649/589637
Model 1—Sepsis (adjusted for sociodemographic factors)	1.00 (Ref)	16.35 (14.16–18.86)
Model 2 (adjusted for sociodemographic factors and health status)	1.00 (Ref)	7.40 (6.32–8.67)
Model 3 (adjusted for sociodemographic factors, health status, and lifestyle score)	1.00 (Ref)	7.30 (6.23–8.55)
Cancer-related mortality		
Events/person-years	2453/4732572	2365/603361
Model 1—Sepsis (adjusted for sociodemographic factors)	1.00 (Ref)	8.55 (8.06–9.07)
Model 2 (adjusted for sociodemographic factors and health status)	1.00 (Ref)	1.77 (1.66–1.87)
Model 3 (adjusted for sociodemographic factors, health status, and lifestyle score)	1.00 (Ref)	1.76 (1.66–1.87)
Infection-related mortality		
Events/person-years	58/4716416	190/586298
Model 1—Sepsis (adjusted for sociodemographic factors)	1.00 (Ref)	28.25 (20.84–38.30)
Model 2 (adjusted for sociodemographic factors and health status)	1.00 (Ref)	9.90 (7.09–13.84)
Model 3 (adjusted for sociodemographic factors, health status, and lifestyle score)	1.00 (Ref)	9.75 (6.97–13.62)
Other-cause mortality		
Events/person-years	539/4719756	299/587235
Model 1—Sepsis (adjusted for sociodemographic factors)	1.00 (Ref)	4.77 (4.11–5.53)
Model 2 (adjusted for sociodemographic factors and health status)	1.00 (Ref)	4.37 (3.69–5.17)
Model 3 (adjusted for sociodemographic factors, health status, and lifestyle score)	1.00 (Ref)	4.32 (3.65–5.11)

Model 1 was adjusted for age at baseline, gender, ethnicity, education, Townsend deprivation index, BMI at baseline. Model 2 was adjusted for age at baseline, gender, ethnicity, education, Townsend deprivation index, BMI at baseline, CVD, diabetes mellitus, Charlson comorbidity index. Model 3 was age at baseline, gender, ethnicity, education, Townsend deprivation index, BMI at baseline, CVD, diabetes mellitus, Charlson comorbidity index, lifestyle score.

**Table 5:** Event rates and adjusted hazard ratio for all-cause and cause-specific premature mortality (death before age 75 years), comparing any sepsis to participants without sepsis.

Model	No sepsis (ref)	Any sepsis
All-cause mortality		
Events/person-years	9008/4754324	9350/625223
Model 1—Sepsis (adjusted for sociodemographic factors)	1.00 (Ref)	6.66 (6.46–6.87)
Model 2 (adjusted for sociodemographic factors and health status)	1.00 (Ref)	2.46 (2.38–2.54)
Model 3 (adjusted for sociodemographic factors, health status, and lifestyle score)	1.00 (Ref)	2.45 (2.37–2.53)
Cardiovascular mortality		
Events/person-years	3641/4714577	3014/570789
Model 1—Sepsis (adjusted for sociodemographic factors)	1.00 (Ref)	4.70 (4.47–4.94)
Model 2 (adjusted for sociodemographic factors and health status)	1.00 (Ref)	2.40 (2.27–2.54)
Model 3 (adjusted for sociodemographic factors, health status, and lifestyle score)	1.00 (Ref)	2.38 (2.25–2.52)
Respiratory mortality		
Events/person-years	527/4689683	1274/553934
Model 1—Sepsis (adjusted for sociodemographic factors)	1.00 (Ref)	14.51 (13.05–16.14)
Model 2 (adjusted for sociodemographic factors and health status)	1.00 (Ref)	7.53 (6.71–8.46)
Model 3 (adjusted for sociodemographic factors, health status, and lifestyle score)	1.00 (Ref)	7.43 (6.62–8.35)
Cancer-related mortality		
Events/person-years	3939/4714450	4082/577961
Model 1—Sepsis (adjusted for sociodemographic factors)	1.00 (Ref)	7.31 (6.98–7.66)
Model 2 (adjusted for sociodemographic factors and health status)	1.00 (Ref)	1.78 (1.70–1.87)
Model 3 (adjusted for sociodemographic factors, health status, and lifestyle score)	1.00 (Ref)	1.78 (1.70–1.86)
Infection-related mortality		
Events/person-years	131/4686685	442/547389
Model 1—Sepsis (adjusted for sociodemographic factors)	1.00 (Ref)	21.58 (17.63–26.41)
Model 2 (adjusted for sociodemographic factors and health status)	1.00 (Ref)	8.81 (7.09–10.95)
Model 3 (adjusted for sociodemographic factors, health status, and lifestyle score)	1.00 (Ref)	8.75 (7.04–10.87)
Other-cause mortality		
Events/person-years	770/4691545	538/548326
Model 1—Sepsis (adjusted for sociodemographic factors)	1.00 (Ref)	5.11 (4.55–5.74)
Model 2 (adjusted for sociodemographic factors and health status)	1.00 (Ref)	4.65 (4.09–5.30)
Model 3 (adjusted for sociodemographic factors, health status, and lifestyle score)	1.00 (Ref)	4.62 (4.05–5.26)

Model 1 was adjusted for age at baseline, gender, ethnicity, education, Townsend deprivation index, BMI at baseline. Model 2 was adjusted for age at baseline, gender, ethnicity, education, Townsend deprivation index, BMI at baseline, CVD, diabetes mellitus, Charlson comorbidity index. Model 3 was age at baseline, gender, ethnicity, education, Townsend deprivation index, BMI at baseline, CVD, diabetes mellitus, Charlson comorbidity index, lifestyle score.

The aHRs for all-cause and cause-specific premature mortality were elevated across all sepsis subtypes—implicit sepsis alone, explicit sepsis alone, and both implicit and explicit sepsis when compared with non-sepsis, with the magnitude of risk varying by sepsis subtype (Table 6). The highest risk of premature mortality was observed in participants with implicit sepsis alone or both implicit and explicit sepsis, followed by those with explicit sepsis alone (Table 6). For example, the aHRs for all-cause mortality were 1.72 (95% CI 1.21–2.45) for implicit sepsis, 2.05 (95% CI 1.94–2.17) for explicit sepsis, and 2.60 (95% CI 2.48–2.73) for both implicit and explicit sepsis.

**Table 6:** Event rates and adjusted hazard ratio for all-cause and cause-specific premature mortality, comparing any subtype sepsis to participants without sepsis.

Model	No sepsis (ref)	Explicit alone	Implicit alone	Both implicit and explicit
All-cause mortality				
Events/person-years	5487/4754324	31/8666	1865/285305	3096/331252
Model 1—Sepsis (adjusted for sociodemographic factors)	1.00 (Ref)	4.00 (2.81–5.70)	6.10 (5.78–6.43)	8.46 (8.08–8.85)
Model 2 (adjusted for sociodemographic factors and health status)	1.00 (Ref)	1.74 (1.22–2.47)	2.06 (1.95–2.18)	2.62 (2.50–2.75)
Model 3 (adjusted for sociodemographic factors, health status, and lifestyle score)	1.00 (Ref)	1.72 (1.21–2.45)	2.05 (1.94–2.17)	2.60 (2.48–2.73)
Cardiovascular mortality				
Events/person-years	2138/4731578	15/8508	608/275716	866/313148
Model 1—Sepsis (adjusted for sociodemographic factors)	1.00 (Ref)	4.80 (2.89–7.98)	4.52 (4.12–4.96)	5.27 (4.85–5.72)
Model 2 (adjusted for sociodemographic factors and health status)	1.00 (Ref)	2.69 (1.62–4.47)	2.22 (2.01–2.45)	2.50 (2.29–2.73)
Model 3 (adjusted for sociodemographic factors, health status, and lifestyle score)	1.00 (Ref)	2.67 (1.61–4.45)	2.20 (2.00–2.43)	2.47 (2.26–2.70)
Respiratory mortality				
Events/person-years	299/4718102	1/8382	224/272284	424/308971
Model 1—Sepsis (adjusted for sociodemographic factors)	1.00 (Ref)	2.23 (0.31–15.91)	12.58 (10.53–15.03)	19.9 (17.06–23.23)
Model 2 (adjusted for sociodemographic factors and health status)	1.00 (Ref)	1.23 (0.17–8.76)	5.90 (4.88–7.13)	8.81 (7.44–10.42)
Model 3 (adjusted for sociodemographic factors, health status, and lifestyle score)	1.00 (Ref)	1.21 (0.17–8.61)	5.83 (4.82–7.04)	8.68 (7.33–10.28)
Cancer-related mortality				
Events/person-years	2453/4732572	10/8455	841/277065	1514/317841
Model 1—Sepsis (adjusted for sociodemographic factors)	1.00 (Ref)	2.95 (1.59–5.49)	6.73 (6.21–7.29)	10.25 (9.59–10.96)
Model 2 (adjusted for sociodemographic factors and health status)	1.00 (Ref)	0.95 (0.51–1.76)	1.49 (1.38–1.62)	1.98 (1.85–2.11)
Model 3 (adjusted for sociodemographic factors, health status, and lifestyle score)	1.00 (Ref)	0.95 (0.51–1.76)	1.49 (1.38–1.62)	1.98 (1.85–2.11)
Infection-related mortality				
Events/person-years	58/4716416	0/8370	45/271042	145/306886
Model 1—Sepsis (adjusted for sociodemographic factors)	1.00 (Ref)	NA	14.83 (9.97–22.04)	40.61 (29.64–55.65)
Model 2 (adjusted for sociodemographic factors and health status)	1.00 (Ref)	NA	5.47 (3.60–8.33)	13.79 (9.75–19.50)
Model 3 (adjusted for sociodemographic factors, health status, and lifestyle score)	1.00 (Ref)	NA	5.40 (3.54–8.21)	13.56 (9.58–19.19)
Other-cause mortality				
Events/person-years	539/4719756	5/8431	147/271878	147/306926
Model 1—Sepsis (adjusted for sociodemographic factors)	1.00 (Ref)	7.20 (2.98–17.39)	5.20 (4.31–6.27)	4.36 (3.61–5.26)
Model 2 (adjusted for sociodemographic factors and health status)	1.00 (Ref)	6.63 (2.74–16.04)	4.76 (3.89–5.83)	3.97 (3.23–4.87)
Model 3 (adjusted for sociodemographic factors, health status, and lifestyle score)	1.00 (Ref)	6.61 (2.73–15.99)	4.70 (3.84–5.76)	3.92 (3.19–4.82)

Model 1 was adjusted for age at baseline, gender, ethnicity, education, Townsend deprivation index, BMI at baseline. Model 2 was adjusted for age at baseline, gender, ethnicity, education, Townsend deprivation index, BMI at baseline, CVD, diabetes mellitus, Charlson comorbidity index. Model 3 was age at baseline, gender, ethnicity, education, Townsend deprivation index, BMI at baseline, CVD, diabetes mellitus, Charlson comorbidity index, lifestyle score.

A sensitivity analysis excluding participants with cancer at enrolment showed that the associations between sepsis and all-cause and cause-specific premature mortality remained of similar magnitude (Supplementary Table S8). An additional analysis using propensity scores for sepsis indicated that these associations were either slightly attenuated or strengthened but remained statistically significant for premature mortality (Supplementary Table S9). Results from multiple imputation analyses were consistent with the primary findings, with slightly higher hazard ratios across all mortality types (Supplementary Table S10). The Fine–Gray competing risk analysis yielded slightly attenuated but consistent associations between sepsis and cause-specific premature mortality compared with the cause-specific Cox models (Supplementary Table S11). Finally, stratified analyses were performed to assess the potential modifying effects of age, CCI, and lifestyle score on the associations between sepsis and premature mortality, showing that the associations were more pronounced among participants who were older, had more comorbidities, and experienced more unhealthy lifestyle behaviors (Supplementary Table S12).

## Discussion

This large population-based cohort study from the UKB provided the evidence linking sepsis with increased risks of all-cause and cause-specific premature mortality. Our findings indicated that individuals diagnosed with sepsis had a higher risk of premature mortality compared to those without sepsis. This elevated risk was particularly pronounced among those with implicit sepsis alone or a combination of implicit and explicit sepsis. These associations persisted across various causes of death, including cardiovascular disease, respiratory disease, infection, and cancer. Notably, these risks were most significant within the first 5 years post-diagnosis, after which they either stabilized or slightly declined. In addition, the risks associated with sepsis were more pronounced in individuals with older age, a higher CCI, and those with an unhealthy lifestyle, suggesting that these factors may aggravate the impact of sepsis on long-term mortality. Our findings support the global health agenda set by the World Health Organization and Sustainable Development Goal (SDG) 3.4 (30), which aims to reduce premature mortality from both noncommunicable and communicable diseases by one-third by 2030. Given that sepsis contributes substantially to premature deaths through infection-related and long-term organ dysfunction, implementing structured post-sepsis care within national health systems—including dedicated follow-up clinics, monitoring for long-term sequelae, and secondary prevention strategies—could be crucial for reducing sepsis-related premature mortality and advancing these international targets.

Our findings were supported by existing literature that underscores the lasting impact of sepsis on long-term mortality. Previous studies have consistently shown that sepsis is associated with elevated risks of death (31–33). For example, a cohort study found that sepsis survivors had a 22% increased risk of mortality within 1 year of discharge compared to hospitalized patients without sepsis (31). Similarly, a large population-based cohort study of 20,313 admissions from Sweden found that although the HR for all-cause mortality attenuated over survival time, it remained elevated at all intervals: HR of 3.0 (95% CI 2.8–3.2) at 2 to 12 months post-admission, 1.8 to 1.9 between 1 and 5 years, and 1.6 (95% CI 1.5–1.8) at more than 5 years (32). Several studies also indicated that these increased mortality risks are particularly evident in older adults, those with multiple comorbidities and unhealthy lifestyle behaviors (33–35). A multicenter longitudinal cohort study conducted in the United States showed that older age was significantly associated with higher sepsis-related mortality rates. The study reported mortality rates of 6.4% for individuals aged 18 to 44 years, 12.5% for those aged 45 to 64 years, 15.2% for those aged 65 to 74 years, 17.6% for those aged 75 to 84 years, and 20.9% for those aged 85 years and older (33). Moreover, a nationwide prospective registry study of 222,832 Norwegians from 2008 to 2021 showed that sepsis patients with comorbidities had an incrementally higher risk of mortality compared to those without comorbidities. Specifically, compared to sepsis patients without any comorbidities, those with one, two, or three or more comorbidities had adjusted HRs of 1.71 (95% CI 1.69–1.71), 2.12 (95% CI 2.09–2.16), and 2.60 (95% CI 2.54–2.67), respectively (34). A meta-analysis pooling 5 studies involving 2,694 septic patients found that smokers had a significantly higher risk of mortality compared to non-smokers (HR 1.62, 95% CI 1.11–2.37), and this risk was even greater among patients followed for more than 2 months (HR 2.33, 95% CI 1.83–2.96) (35). Additionally, studies have consistently shown that implicit sepsis, which often involves systemic infection without the clinical symptoms of sepsis, was associated with a higher risk of adverse outcomes compared to explicit sepsis, where the diagnosis tends to be clearer and leads to quicker intervention (22, 34). Our findings on the differential effect of implicit and explicit sepsis were consistent with previous studies that suggest implicit sepsis, which is often underdiagnosed and undertreated, may carry a higher mortality risk due to delays in recognition and intervention (22, 34).

Several mechanisms may explain the increased mortality risk associated with sepsis, particularly implicit sepsis. First, the systemic inflammatory response syndrome triggered by sepsis might result in endothelial dysfunction, microvascular thrombosis, and subsequent multi-organ failure, all of which contribute to early and long-term mortality (36). Implicit sepsis may be more likely to go unrecognized or be undertreated due to the absence of overt clinical symptoms associated with explicit sepsis, allowing the underlying infection to progress unchecked (22). Additionally, sepsis is known to induce immune dysregulation, characterized by both hyperinflammation and immune suppression, which can persist long after the acute infection has resolved. The immune suppression or “immune paralysis,” driven in part by T-cell exhaustion and checkpoint pathway activation (PD-1/PD-L1, CTLA-4), undermines adaptive immune competence and predisposes survivors to secondary infection and late mortality (37–39). The dysbiosis of the gut microbiota after sepsis—characterized by reduced diversity, loss of beneficial taxa such as Lentisphaeria, Coprococcus, and Lachnospiraceae FCS020, and overgrowth of potentially harmful genera including Clostridiaceae1, *Eubacterium eligens*, Gordonibacter, and Terrisporobacter—has been linked to heightened systemic inflammation and impaired mucosal immunity, thereby increasing vulnerability to recurrent infections, metabolic disturbances, and malignancy (40). Moreover, the physiological stress of sepsis can exacerbate pre-existing conditions, such as cardiovascular disease or diabetes, leading to deterioration in health and increased mortality risk (41, 42). The more pronounced effects in those with older age and multiple comorbidities, and individuals with unhealthy lifestyles may reflect the interaction between sepsis and these underlying vulnerabilities, further amplifying the risk of poor outcomes.

This study has several strengths. The UKB is a large and well-characterized cohort, allowing us to conduct a detailed analysis of the associations between sepsis and premature mortality across a diverse population. The inclusion of detailed sociodemographic, clinical, and lifestyle data enabled us to control for a wide range of potential confounders, thereby strengthening the causal inferences that can be drawn from our results. Furthermore, the stratified analyses by age, comorbidity, and lifestyle factors provided valuable insights into the differential effect of sepsis across various subgroups, highlighting importance in targeted interventions. Our study also has limitations. First, despite the extensive data available in the UKB, the diagnosis of sepsis was based on ICD-10 codes from hospital records, which may lead to misclassification of sepsis cases. Implicit sepsis, in particular, was challenging to diagnose and may have been underreported, potentially biasing our results. Second, while we adjusted for a wide range of confounders, residual confounding cannot be entirely ruled out, particularly concerning unmeasured factors such as the severity of the initial sepsis episode, access to healthcare, and variations in treatment quality. Additionally, because UK Biobank data do not allow precise timing of sepsis onset, we treated sepsis exposure as fixed, which could introduce some immortal-time bias; however, the long follow-up and consistent sensitivity analyses suggest minimal impact on our conclusions. Third, the UK Biobank cohort, although large sample size, is not entirely representative of the general population, particularly in terms of ethnic diversity and socioeconomic status, which may lead to underestimation of sepsis-related mortality in vulnerable populations and limits generalizability to low- and middle-income settings. In addition, as our study was conducted solely within the UK population, the generalizability to other non-UK populations, such as the Chinese population, remains uncertain and warrants confirmation in future studies. Fourth, because our analysis was restricted to hospitalized participants and conducted over more than 10 years of follow-up, the observed proportion of sepsis reflects cumulative incidence and may overestimate the absolute burden compared with the general population. Fifth, sepsis subtypes were not available in the UK Biobank data, precluding us from examining potential heterogeneity across different forms of sepsis. Our findings should be interpreted with caution, and further studies with detailed subtype information are needed to determine differences in premature mortality risk across sepsis subgroups. Sixth, although diagnostic and coding variability may lead to some misclassification between explicit and implicit sepsis, our analyses showed consistent associations across all septic subgroups, indicating the robustness of the observed associations. Seventh, excluded participants were older and had less favorable health profiles than those included (Supplementary Table S13), suggesting that exclusion due to missing data may have slightly attenuated the observed associations. Nevertheless, the consistency of results across complete-case, multiple imputation, and propensity-score analyses supports the robustness of our conclusions. Lastly, the observational nature of our study precludes definitive conclusions about causality. Future studies using target trial emulation (43) and Mendelian Randomization, alongside complementary statistical methods such as marginal structural models, inverse probability weighting, could help reduce residual confounding, approximate causal effects more reliably, and provide more robust evidence to inform clinical decision-making.

## Conclusion

Sepsis, particularly implicit and combined implicit-explicit sepsis, was associated with significantly increased risks of all-cause and cause-specific premature mortality. The risks were especially pronounced within the first 5 years’ post-diagnosis and were further amplified in individuals with older age and multiple comorbidities, and those with unhealthy lifestyles. These findings highlighted the urgent need for early recognition and aggressive management of sepsis, especially in high-risk populations, to improve long-term survival outcomes. Further research should focus on developing targeted interventions and long-term care strategies for sepsis survivors to mitigate these elevated mortality risks.

## Data Availability

The datasets presented in this article are not readily available because data will be made available on request. Requests to access the datasets should be directed to Lei Yuan, yuanleigz@smmu.edu.cn.
