# Supplementary material for: Association between sepsis and all-cause and cause-specific premature mortality: a prospective cohort study
Source: Front Public Health. 2025 Nov 14;13:1666675. doi: 10.3389/fpubh.2025.1666675 (PMC12660079; doi:10.3389/fpubh.2025.1666675)
Supplement: Supplementary file 1 [file Data_Sheet_1.PDF]

Sepsis with all-cause and cause-specific premature mortality

Design

Prospective cohort
N= 371558

Participants
Aged 39~71 years

Median follow-up
15.0 years

Exposure

Type and number of sepsis—ICD-10 codes

Explicit alone	Implicit alone	Implicit and explicit
620 (0.2%)	21148 (5.7%)	25381 (6.8%)

Outcome

Premature death before the age of 70—death certificate

Cause of death

All-cause	CVD	Infection	Respiratory	Other
N=10479	N=3627	N=248	N=948	N=838

CVD: cardiovascular disease

Results

HR (95% CI) for premature mortality by type of sepsis

	All-cause mortality	Cardiovascular mortality
No sepsis	1.00 (ref.)	1.00 (ref.)
Any sepsis	**2.36 (2.26–2.46)**	**2.35 (2.18–2.54)**
Explicit alone	**1.72 (1.21–2.45)**	**2.67 (1.61–4.45)**
Implicit alone	**2.05 (1.94–2.17)**	**2.20 (2.00–2.43)**
Implicit and explicit	**2.60 (2.48–2.73)**	**2.47 (2.26–2.70)**

CI: confidence interval

Premature mortality rates beginning with diagnosis among individuals with sepsis and without sepsis

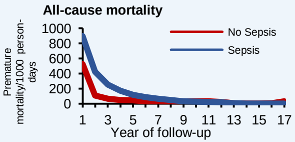

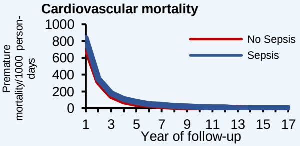

Conclusions

Sepsis, especially implicit and combined implicit-explicit sepsis, was associated with increased risks of all-cause and cause-specific premature mortality.
