# Supplementary material for: Association between sepsis and all-cause and cause-specific premature mortality: a prospective cohort study
Source: Front Public Health. 2025 Nov 14;13:1666675. doi: 10.3389/fpubh.2025.1666675 (PMC12660079; doi:10.3389/fpubh.2025.1666675)
Supplement: Supplementary file 2 [file Table_1.DOCX]

**Association between sepsis and all-cause and cause-specific premature mortality: a prospective cohort study**

**Supplementary Method 1. Lifestyle score calculation.**

Lifestyle factors were investigated, including smoking status, alcohol consumption, physical activity, television viewing time, sleep duration, and diet quality. For smoking status, participants were classified into never, former, and current smokers. For alcohol consumption, the number of drinks per week was computed by summing average weekly consumption of various types of alcohol, including champagne, white wine, beer, and others. For physical activity, walking, moderate and vigorous activity were measured according to the validated short International Physical Activity Questionnaire scoring protocol and summed as metabolic equivalents (MET-hours/week). For dietary quality assessment, participants were asked to complete a validated questionnaire that included questions on frequency of food consumption (fruit and vegetables intake, oily fish intake, red meat intake, and processed meat intake) over the past year. In order to provide a composite assessment for lifestyle factors, a previously published lifestyle scoring system derived from an Australian cohort was used. Briefly, the scoring system dichotomized each lifestyle variable as 0 points if not at risk or 1 point if at risk, and summed to create an unweighted lifestyle score. The lifestyle score ranged from 0 to 9, with higher values indicated an unhealthier lifestyle. Participants were classified into three categories according to their lifestyle score, with those who scored 0–2 were classed as most healthy, those who scored 3–5 were classed as moderately healthy, and those who scored 6–9 were classed as least healthy.

**Supplementary Table 1. Definitions and description of explicit sepsis**

| **Description** | **ICD-10-CM** |
| --- | --- |
| Typhoid fever sepsis | A01.003 |
| Septic shock | A41.9 |
| Salmonella sepsis | A02.100 |
| Shigellosis, unspecified (shock) | A03.900 |
| Septic gastroenteritis | A09.005 |
| Septicemic plague | A20.7 |
| Generalized tularemia | A21.7 |
| Anthrax sepsis | A22.7 |
| Acute and fulminating melioidosis | A24.1 |
| Erysipelothrix sepsis | A26.7 |
| Leptospirosis | A27.900 |
| Pasteurellosis | A28.001 |
| Extraintestinal yersiniosis | A28.2 |
| Listerial sepsis | A32.7 |
| Scarlet fever sepsis | A38.x00x012 |
| Acute meningococcemia | A39.2 |
| Chronic meningococcemia | A39.3 |
| Meningococcemia， unspecified | A39.4 |
| Waterhouse | A39.1 |
| Streptococcal sepsis | A40 |
| Other sepsis | A41 |
| Actinomycotic sepsis | A42.7 |
| Toxic shock syndrome | A48.3 |
| Other gonococcal infections (sepsis) | A54.8 |
| Other specified arthropod-borne viral fevers [Piry virus disease] | A93.800x001 |
| Haemorrhagic fever with renal syndrome | A98.500 |
| Herpetic septicemia | B00.7 |
| Candidal sepsis | B37.7 |
| Candidal endocarditis | B37.6 |
| Unspecified mycosis (Fungemia) | B49 |
| Delirium, unspecified (infectious) | F05.901 |
| Other specified mental disorders due to brain damage and dysfunction and to physical disease (biliary infection) | F06.800x002 |
| Other specified mental disorders due to brain damage and dysfunction and to physical disease (bacillary dysentery) | F06.800x016 |
| Bacterial pneumonia, unspecified (severe community-acquired) | J15.903 |
| Pneumonia, unspecified (severe) | J18.903 |
| Sepsis of tracheostomy stoma | J95.000x001 |
| Abscess of pancreas (severe) | K85.800x019 |
| Abscess of pancreas (other, severe) | K85.817 |
| Spontaneous abortion, Incomplete, with septic shock | O03.300x001 |
| Spontaneous abortion, Complete, with septic shock | O03.800x001 |
| Medical abortion, Incomplete, with septic shock | O04.300x004 |
| Medical abortion, Complete, with septic shock | O04.800x001 |
| Medical abortion, later complete, with septic shock | O04.804 |
| Genital tract and pelvic infection following abortion and ectopic and molar pregnancy | O08.000 |
| Septic shock following abortion and ectopic and molar pregnancy | O08.003 |
| Embolism following abortion and ectopic and molar pregnancy (septic) | O08.200x002 |
| Embolism following abortion and ectopic and molar pregnancy (septicopyaemic) | O08.200x006 |
| Other infection during labour | O75.3 |
| Puerperal sepsis | O85 |
| Obstetric pyaemic and septic embolism | O88.300 |
| Other maternal infectious and parasitic diseases complicating pregnancy, childbirth and the puerperium | O98.8 |
| Bacterial sepsis of newborn | P36 |
| Other specified congenital infectious and parasitic diseases | P37.800x002 |
| Septic shock | R57.2 |
| Systemic inflammatory response syndrome (SIRS) due to infection with organ dysfunction | R65.1 |
| Infections following infusion, transfusion and therapeutic injection | T80.2 |
| Infection following a procedure, not elsewhere classified | T81.4 |
| Sepsis following immunization | T88.000x002 |

**Supplementary Table 2. Definitions and description of infection and sepsis-related death**

| **Description** | **ICD-10-CM** |
| --- | --- |
| Cholera | A00 |
| Typhoid/paratyphoid fever | A01 |
| Other salmonella infection | A02, G01, J17, M01, M90 |
| Shigellosis | A03 |
| Other food poisoning | A05 |
| Intestinal infection not otherwise classified | A02, A04, A08 |
| Ill-defined intestinal infection | A09 |
| Primary tuberculosis | A15, A16 |
| Pulmonary tuberculosis | A15, A16 |
| Other respiratory tuberculosis | A15, A16, J38 |
| Central nervous system tuberculosis | A17 |
| Intestinal tuberculosis | A18, K93 |
| Tuberculosis of bone and joint | A18, H75 |
| Genitourinary tuberculosis | A18, N51, N74 |
| Tuberculosis not otherwise classified | A18, D77, E35, K23 |
| Military tuberculosis | A19 |
| Plague | A20 |
| Tularemia | A21 |
| Anthrax | A22 |
| Brucellosis | A23 |
| Glanders | A24 |
| Melioidosis | A24 |
| Rat-bite fever | A25 |
| Other bacterial zoonoses | A26, A28, A32 |
| Leprosy | A30 |
| Other mycobacterial disease | A31 |
| Diphtheria | A36, I41, K67, N33 |
| Whooping cough | A37 |
| Streptococcal throat/scarlet fever | A38, J02 |
| Erysipelas | A46 |
| Meningococcal infection | A39, G05, H48, M01 |
| Tetanus | A35 |
| Septicemia | A40, A41 |
| Actinomycotic infections | A42, A43, B47 |
| Other bacterial diseases | A48, K90, M60 |
| Bacterial infection in other diseases not otherwise specified | B95, B96 |
| Congenital syphilis | A50 |
| Early symptomatic syphilis | A51, R59, H22, H32, M90, K77, G01, L99 |
| Early syphilis latent | A51 |
| Cardiovascular syphilis | A52, I32, I39, I41, I79 |
| Neurosyphilis | A52, G01, G05, H32, H48, H94, I60 |
| Other late symptomatic syphilis | A52, H19, J99, K67, K77, N29, M63, M68 , M90 |
| Late syphilis latent | A52 |
| Other and unspecified syphilis | A52, A53 |
| Gonococcal infections | A54, N30, N33, N39, N51, N72, N74, H19, G01, I32, I39, I52, K67 |
| Leptospirosis | A27, G01 |
| Vincent’s angina | A69 |
| Yaws | A66 |
| Pinta | A67 |
| Other spirochetal infection | A65, A69 |
| Dermatophytosis | B35 |
| Dermatomycosis not otherwise classified or specified | B36 |
| Candidiasis | A09, B37, H60 |
| Coccidioidomycosis | B38 |
| Histoplasmosis | B39, G02, H36, I32, I39, J99 |
| Blastomycotic infection | B40, B41, B48 |
| Other mycoses | B42, B43, B44, B45, B46, B47, B48 |
| Opportunistic mycoses | B48 |
| Bacterial meningitis | G00 |
| Meningitis, unspecified | G03, G05 |
| Central nervous system abscess | G06 |
| Phlebitis of intracranial sinus | G08 |
| Acute pericarditis | I30, I32 |
| Acute or subacute endocarditis | I33, I39 |
| Thrombophlebitis | I80 |
| Acute sinusitis | J01 |
| Acute pharyngitis | J02 |
| Acute tonsillitis | J03 |
| Acute laryngitis/tracheitis | J04, J05 |
| Acute upper respiratory infection of multiple sites/not otherwise specified | J06 |
| Pneumococcal pneumonia | J13 |
| Other bacterial pneumonia | J14, J15 |
| Bronchopneumonia with organism not otherwise specified | J18 |
| Pneumonia, organism not otherwise specified | J18 |
| Acute exacerbation of obstructive chronic bronchitis | J44.1 |
| Bronchiectasis | J47 |
| Empyema | J86 |
| Lung/mediastinum abscess | J85 |
| Acute appendicitis | K35 |
| Appendicitis not otherwise specified | K37 |
| Other appendicitis | K36 |
| Diverticulitis of small intestine without hemorrhage | K57.12 |
| Diverticulitis of small intestine with hemorrhage | K57.13 |
| Diverticulitis of colon without hemorrhage | K57.22 |
| Diverticulitis of colon with hemorrhage | K57.23 |
| Anal and rectal abscess | K61 |
| Peritonitis | K65, K67 |
| Intestinal abscess | K63.0 |
| Perforation of intestine | K63.1 |
| Abscess of liver | K75.0 |
| Portal pyema | K75.1 |
| Acute cholecystitis | K81.0 |
| Kidney infection | N10, N11, N12, N15 |
| Urethritis/urethral syndrome | N34 |
| Urinary tract infection not otherwise specified | N39.0 |
| Prostatic inflammation | N41, N51 |
| Female pelvic inflammation disease | N70 |
| Uterine inflammation disease | N71 |
| Other female genital inflammation | N72, N75, N76, N77 |
| Cellulitis, finger/toe | L03 |
| Other cellulitis or abscess | L03 |
| Acute lymphadenitis | L04 |
| Other local skin infection | L08, L98 |
| Pyogenic arthritis | M00.9 |
| Osteomyelitis | M86, M89, M90 |
| Bacteremia | A49.9 |
| Infection or inflammation of device/graft | T82.6, T82.7, T83.5, T84.5, T84.6, T85.71, T85.78, T85.81 |
| Postoperative infection | T81.4 |
| Infectious complication of medical care not otherwise classified | T80.2 |
| Bacterial infection of unspecified site | A49 |
| Typhus fever | A75 |
| Spotted fever [tick-borne rickettsioses] | A77 |
| Q fever | A78 |
| Other rickettsioses | A79 |
| Acute poliomyelitis | A80 |
| Atypical virus infections of central nervous system | A81 |
| Rabies | A82 |
| Mosquito-borne viral encephalitis | A83 |
| Tick-borne viral encephalitis | A84 |
| Other viral encephalitis, not elsewhere classified | A85 |
| Unspecified viral encephalitis | A86 |
| Viral meningitis | A87 |
| Other viral infections of central nervous system, not elsewhere classified | A88 |
| Unspecified viral infection of central nervous system | A89 |
| Dengue fever [classical dengue] | A90 |
| Dengue hemorrhagic fever | A91 |
| Other mosquito-borne viral fevers | A92 |
| Other arthropod-borne viral fevers, not elsewhere classified | A93 |
| Unspecified arthropod-borne viral fever | A94 |
| Yellow fever | A95 |
| Arenaviral hemorrhagic fever | A96 |
| Other viral hemorrhagic fevers, not elsewhere classified | A98 |
| Unspecified viral hemorrhagic fever | A99 |
| Other viral diseases, not elsewhere classified | B33 |
| Viral infection of unspecified site | B34 |
| Unspecified mycosis | B49 |
| Plasmodium falciparum malaria | B50 |
| Plasmodium vivax malaria | B51 |
| Plasmodium malariae malaria | B52 |
| Other specified malaria | B53 |
| Unspecified malaria | B54 |
| Leishmaniasis | B55 |
| African trypanosomiasis | B56 |
| Chagas' disease | B57 |
| Toxoplasmosis | B58 |
| Pneumocystosis | B59 |
| Other protozoal diseases, not elsewhere classified | B60 |
| Unspecified protozoal disease | B64 |
| Schistosomiasis [bilharziasis] | B65 |
| Other fluke infections | B66 |
| Echinococcosis | B67 |
| Taeniasis | B68 |
| Cysticercosis | B69 |
| Diphyllobothriasis and sparganosis | B70 |
| Other cestode infections | B71 |
| Dracunculiasis | B72 |
| Onchocerciasis | B73 |
| Filariasis | B74 |
| Trichinellosis | B75 |
| Hookworm diseases | B76 |
| Ascariasis | B77 |
| Strongyloidiasis | B78 |
| Trichuriasis | B79 |
| Enterobiasis | B80 |
| Other intestinal helminthiases, not elsewhere classified | B81 |
| Unspecified intestinal parasitism | B82 |
| Other helminthiases | B83 |
| Pediculosis and phthiriasis | B85 |

**Supplementary Table 3. Definitions and description of organ dysfunction**

| **Description** | **ICD-10-CM** |
| --- | --- |
| Septic shock | A41.9 |
| Toxic shock syndrome | A48.3 |
| Volume depletion | E86.x00 |
| hypovolemia | E86.x00x001 |
| extracellular fluid deletion | E86.x00x003 |
| Plasma volume depletion | E86.x00x004 |
| Volume depletion | E86.x00x005 |
| dehydration | E86.x01 |
| Severe Myocarditis | I51.400x007 |
| Hypotension, unspecified | I95.8 |
| Hypotension, | I95.9 |
| Other disorders of the circulatory system | I99.x00 |
| Circulatory disorders | I99.x01 |
| Weak pulse | R09.800x082 |
| Cardiogenic shock | R57.0 |
| Hypovolaemic shock | R57.1 |
| Septic shock | R57.2 |
| Other shock | R57.8 |
| Shock, unspecified | R57.9 |
| Spontaneous abortion, Incomplete, with septic shock | O03.300x001 |
| Spontaneous abortion, Complete, with septic shock | O03.800x001 |
| Medical abortion, Incomplete, with septic shock | O04.300x004 |
| Medical abortion, Complete, with septic shock | O04.800x001 |
| Medical abortion, later complete, with septic shock | O04.804 |
| Cardiovascular disorders originating in the perinatal period | P29 |
| Acute respiratory distress syndrome | J80 |
| Pulmonary oedema | J81 |
| Acute pulmonary insufficiency after thoracic surgery | J95.100 |
| Acute pulmonary insufficiency after non-thoracic surgery | J95.200 |
| Respiratory failure after surgical procedures | J95.800x004 |
| Respiratory distress syndrome in adults after surgery | J95.800x021 |
| Acute respiratory failure with hypoxia, not elsewhere classified | J96 |
| Respiratory failure, unspecified with hypoxia | J96.9 |
| Other disorders of lung | J98.4 |
| Severe infection of the lungs | J98.400x024 |
| Respiratory distress of newborn | P22 |
| Respiratory failure of newborn | P28.5 |
| Asphyxia | R09.0 |
| suffocate | R09.000 |
| Respiratory arrest | R09.2 |
| Asthma | R09.800x095 |
| Severe acute respiratory syndrome [SARS], unspecified | U04.9 |
| Delirium (not alcohol- or drug-induced) | F05 |
| Other specified mental disorders due to infection | F06.8 |
| Anoxic brain damage, not elsewhere classified | G93.1 |
| Encephalopathy, unspecified | G93.4 |
| Other specified disorders of brain | G93.8 |
| Disorder of brain, unspecified | G93.9 |
| Somnolence, stupor and coma | R40 |
| Disorientation, unspecified | R41.0 |
| Demoralization and apathy | R45.3 |
| Syncope and collapse | R55 |
| Haemorrhagic fever with renal syndrome | A98.500 |
| Acute kidney failure | N17 |
| Unspecified kidney failure | N19 |
| Anuria and oliguria | R34 |
| Extrarenal uraemia | R39.2 |
| Abnormal results of kidney function studies | R94.4 |
| Acidosis | E87.2 |
| Other specified arthropod-borne viral fevers [Piry virus disease] | A93.800x001 |
| Bone marrow suppression | D61.900x001 |
| Myelosuppressive anemia | D61.901 |
| Pancytopenia | D61.903 |
| Acute bone marrow hematopoietic function inhibition | D61.906 |
| Disseminated intravascular coagulation [defibrination syndrome] | D65 |
| Gangrene purpura | D65.x00x003 |
| Acquired fibrinogen deficiency | D65.x01 |
| Acquired fibrinolytic bleeding | D65.x02 |
| Fibrinolytic purpura | D65.x03 |
| Coagulation defect, unspecified | D68.9 |
| Infectious purpura | D69.000x008 |
| Bacterial purpura | D69.000x011 |
| Toxic purpura | D69.000x013 |
| purpura | D69.203 |
| Hemorrhagic purpura | D69.301 |
| Secondary thrombocytopenia | D69.5 |
| Secondary thrombocytopenic purpura | D69.501 |
| Thrombocytopenia, unspecified | D69.6 |
| Other specified haemorrhagic conditions | D69.8 |
| Infectious hemophagocytic syndrome | D76.200x001 |
| Infectious erythrophagocytic syndrome | D76.200x011 |
| Hepatitis A, accompanied by hepatic coma | B15.000 |
| Acute viral hepatitis A with hepatic coma | B15.001 |
| Acute severe severe viral hepatitis A with hepatic coma | B15.002 |
| Subacute severe viral hepatitis A with hepatic coma | B15.003 |
| Acute hepatitis B, with δ factor (co-infection) and accompanied by hepatic coma | B16.000 |
| Acute hepatitis B-D with hepatic coma | B16.001 |
| Acute hepatitis B, not accompanied by δ factor (co-infection), but with hepatic coma | B16.200 |
| Acute viral hepatitis B with hepatic coma | B16.201 |
| Subacute severe viral hepatitis B with hepatic coma | B16.202 |
| Acute severe hepatitis B with hepatic coma | B16.203 |
| Acute jaundice-free hepatitis B with hepatic coma | B16.204 |
| Acute severe hepatitis B with hepatic coma | B16.206 |
| Acute severe hepatitis hepatitis | B17.807 |
| Viral hepatitis, accompanied by hepatic coma | B19.000 |
| Viral hepatitis with hepatic coma | B19.000x001 |
| Acute severe viral hepatitis with hepatic coma | B19.001 |
| Subacute severe viral hepatitis with hepatic coma | B19.002 |
| Cytome hepatitis with hepatic coma | B25.101† |
| Bilirubin metabolism disorders, others | E80.600 |
| Hyperbilirubinemia | E80.604 |
| Bilirubin metabolism disorders | E80.700 |
| Toxic liver disease with liver failure | K71.100x001 |
| Toxic liver failure | K71.103 |
| Acute and subacute hepatic failure | K72.0 |
| Hepatic failure, unspecified | K72.9 |
| Hepatorenal syndrome | K76.7 |
| Other specified diseases of liver | K76.8 |
| Liver disease, unspecified | K76.9 |
| Liver failure after surgery | K91.825 |
| Typhoid fever sepsis | A01.003 |
| Salmonella sepsis | A02.100 |
| Shigellosis, unspecified (shock) | A03.900 |
| Septic gastroenteritis | A09.005 |
| Septicemic plague | A20.7 |
| Generalized tularemia | A21.7 |
| Anthrax sepsis | A22.7 |
| Acute and fulminating melioidosis | A24.1 |
| Erysipelothrix sepsis | A26.7 |
| Leptospirosis | A27.900 |
| Pasteurellosis | A28.001 |
| Extraintestinal yersiniosis | A28.2 |
| Listerial sepsis | A32.7 |
| Scarlet fever sepsis | A38.x00x012 |
| Acute meningococcemia | A39.2 |
| Chronic meningococcemia | A39.3 |
| Meningococcemia， unspecified | A39.4 |
| Waterhouse | A39.1 |
| Streptococcal sepsis | A40. |
| Other sepsis | A41. |
| Actinomycotic sepsis | A42.7 |
| Streptococcal infection syndrome | A49.103 |
| Other gonococcal infections (sepsis) | A54.8 |
| Polio-like syndrome | A88.800x001 |
| Herpetic septicemia | B00.7 |
| Candidal sepsis | B37.7 |
| Candidal endocarditis | B37.6 |
| Unspecified mycosis (Fungemia) | B49 |
| Progressive septic granulomatous disease | D71.x00x005 |
| Bacterial pneumonia, unspecified (severe community-acquired) | J15.903 |
| Pneumonia, unspecified (severe) | J18.903 |
| Sepsis of tracheostomy stoma | J95.000x001 |
| Sepsis following abortion and ectopic and molar pregnancy | O08.000x006 |
| Embolism following abortion and ectopic and molar pregnancy (septic) | O08.200x002 |
| Embolism following abortion and ectopic and molar pregnancy (septicopyaemic) | O08.200x006 |
| Puerperal sepsis | O85 |
| Obstetric pyaemic and septic embolism | O88.300 |
| Bacterial sepsis of newborn | P36 |
| Other specified congenital infectious and parasitic diseases | P37.800x002 |
| Involves other specific signs and symptoms of the circulatory and respiratory systems | R09.800 |
| Systemic inflammatory response syndrome (SIRS) due to infection with organ dysfunction | R65.1 |
| Infections following infusion, transfusion and therapeutic injection | T80.2 |
| Infection following a procedure, not elsewhere classified | T81.4 |
| Sepsis following immunization | T88.000x002 |
| Multiple organ failure | R68.800x001 |

**Supplementary Table 4. Variables used to create lifestyle score and scoring system for UK Biobank**

| **Lifestyle factor** |  |  |  |  |
| --- | --- | --- | --- | --- |
|  | UK Biobank | | |  |
|  | Questionnaire | ‘Healthy’ | ‘Unhealthy’ |  |
| **Smoking status** | Do you smoke tobacco now? and "In the past, how often have you smoked tobacco?" | past or never smoker | current |  |
| **Alcohol intake** | “About how often do you drink alcohol?” | ≤ 4 times week | Daily or almost daily |  |
| **Physical activity** | IPAQ short form2– total time walking or moderate and vigorous-intensity PA in previous week * | ≥150 min/week moderate or ≥ 75 min/week vigorous PA | <150 min/week moderate or < 75 min/week vigorous PA |  |
| **TV viewing /sedentary time** | “In a typical day, how many hours do you spend watching TV?” | < 4 h/day | ≥ 4 h/day |  |
| **Sleep time** | “About how many hours sleep do you get in every 24 hours?” | >7 or <9h/day | <7 or >9h/day |  |
| **Dietary characteristics** | Individual dietary components contributed directly to lifestyle score. | | |  |
| **Fruit and vegetable intake** | "About how many of …. would you eat per day?” Separate questions for pieces of fresh and dried fruit, tablespoons of salad or cooked/raw vegetables. Combined and converted to g/day (1 portion = 80 g) | ≥ 400 g/day | <400 g/ day |  |
|  |  |  |  |  |
| **Oily fish intake** | "How often do you eat oily fish? (e.g. sardines, salmon, mackerel, herring)" | ≥1 portion/week | <1 portion/week |  |
| **Red meat intake** | "How often do you eat…?” Separate questions for Beef / lamb or mutton / pork (excluding processed meats such as ham or bacon). Red meat included due to clear link between red meat and mortality.3 | ≤3 portion/week | >3 portion/week |  |
| **Processed meat intake** | "How often do you eat processed meats (such as bacon, ham, sausages, meat pies, kebabs, burgers, chicken nuggets)?" | ≤1 portion/week | >1 portion/week |  |

**Supplementary Table 5. Definitions and description of Charlson comorbidity index**

| **Description** | **ICD-10-CM** |
| --- | --- |
| Myocardial infarction | I21.x, I22.x, I25.2 |
| Congestive heart failure | I09.9, I11.0, I13.0, I13.2, I25.5, I42.0, I42.5 - I42.9, I43.x, I50.x, P29.0 |
| Peripheral vascular disease | I70.x, I71.x, I73.1, I73.8, I73.9, I77.1, I79.0, I79.2, K55.1, K55.8, K55.9, Z95.8, Z95.9 |
| Cerebrovascular disease | G45.x, G46.x, H34.0, I60.x - I69.x |
| Dementia | F00.x - F03.x, F05.1, G30.x, G31.1 |
| Chronic pulmonary disease | I27.8, I27.9, J40.x - J47.x, J60.x - J67.x, J68.4, J70.1, J70.3 |
| Rheumatic disease | M05.x, M06.x, M31.5, M32.x - M34.x, M35.1, M35.3, M36.0 |
| Peptic ulcer disease | K25.x - K28.x |
| Mild liver disease | B18.x, K70.0 - K70.3, K70.9, K71.3 - K71.5, K71.7, K73.x, K74.x, K76.0, K76.2 - K76.4, K76.8, K76.9, Z94.4 |
| Diabetes without chronic complication | E10.0, E10.1, E10.6, E10.8, E10.9, E11.0, E11.1, E11.6, E11.8, E11.9, E12.0, E12.1, E12.6, E12.8, E12.9, E13.0, E13.1, E13.6, E13.8, E13.9, E14.0, E14.1, E14.6, E14.8, E14.9 |
| Diabetes with chronic complication | E10.2 - E10.5, E10.7, E11.2 - E11.5, E11.7, E12.2 - E12.5, E12.7, E13.2 - E13.5, E13.7, E14.2 - E14.5, E14.7 |
| Hemiplegia or paraplegia | G04.1, G11.4, G80.1, G80.2, G81.x, G82.x, G83.0 - G83.4, G83.9 |
| Renal disease | I12.0, I13.1, N03.2 - N03.7, N05.2 - N05.7, N18.x, N19.x, N25.0, Z49.0 - Z49.2, Z94.0, Z99.2 |
| Any malignancy, including lymphoma and leukaemia, except malignant neoplasm of skin | C00.x - C26.x, C30.x - C34.x, C37.x - C41.x, C43.x, C45.x - C58.x, C60.x - C76.x, C81.x - C85.x, C88.x, C90.x - C97.x |
| Metastatic cancer | C77, C78, C79, C80 |
| Moderate or severe liver disease | I85.0, I85.9, I86.4, I98.2, K70.4, K71.1, K72.1, K72.9, K76.5, K76.6, K76.7 |
| Acquired immune deficiency syndrome | B20, B21, B22, B20, B23, B24 |

**Supplementary Table 6. Results of the Schoenfeld residual test**

| **Variable** | **rho** | ***χ²* statistic** | ***P* value** |
| --- | --- | --- | --- |
| Sepsis | 0.042 | 1.27 | 0.259 |
| Age at baseline | –0.038 | 0.96 | 0.327 |
| Gender | –0.021 | 0.54 | 0.462 |
| Ethnicity | 0.033 | 0.81 | 0.369 |
| Education | –0.028 | 0.67 | 0.412 |
| Townsend deprivation index | 0.017 | 0.33 | 0.566 |
| BMI at baseline | 0.044 | 1.35 | 0.246 |
| Cardiovascular disease | –0.041 | 1.18 | 0.278 |
| Diabetes mellitus | –0.030 | 0.84 | 0.36 |
| Charlson comorbidity index | 0.039 | 1.04 | 0.308 |
| Lifestyle score | –0.036 | 0.92 | 0.338 |

**Supplementary Table 7. Variables included in the propensity score for sepsis and standard mean differences between with and without sepsis groups before and after propensity score overlap weighting**

| **Model** | **Before Propensity Score Weighting** | | | **After Propensity Score Weighting** | | |
| --- | --- | --- | --- | --- | --- | --- |
|  | No sepsis | Sepsis | Standardized Difference | No sepsis | Sepsis | Standardized  Difference |
| **Gender** |  |  |  |  |  |  |
| Female | 53.4 | 44.8 | 1.053 | 47.0 | 47.0 | 0.000 |
| Male | 46.6 | 55.2 | 0.861 | 53.0 | 53.0 | 0.000 |
| **Age group** |  |  |  |  |  |  |
| ≤54 | 43.0 | 21.8 | 0.962 | 25.3 | 25.3 | 0.000 |
| 55-59 | 18.5 | 15.8 | 0.508 | 16.8 | 16.8 | 0.000 |
| 60-64 | 23 | 29.2 | 0.523 | 28.7 | 28.7 | 0.000 |
| ≥65 | 15.5 | 33.3 | 0.333 | 29.2 | 29.2 | 0.000 |
| **Ethnicity** |  |  |  |  |  |  |
| White | 95.4 | 95.9 | 2.029 | 95.8 | 95.8 | 0.000 |
| Non-white | 4.6 | 4.1 | 0.236 | 4.2 | 4.2 | 0.000 |
| **Education** |  |  |  |  |  |  |
| College or University degree | 13.1 | 10.6 | 0.422 | 11.1 | 11.1 | 0.000 |
| Others | 86.9 | 89.4 | 1.713 | 88.9 | 88.9 | 0.000 |
| **Townsend deprivation index quintile** |  |  |  |  |  |  |
| 1 (least deprived) | 20.5 | 17.3 | 0.540 | 18.2 | 18.2 | 0.000 |
| 2 | 20.1 | 18.4 | 0.526 | 19 | 19.00 | 0.000 |
| 3 | 20.2 | 19.1 | 0.523 | 19.4 | 19.4 | 0.000 |
| 4 | 20 | 20 | 0.514 | 20.1 | 20.1 | 0.000 |
| 5 (most deprived) | 19.2 | 25.1 | 0.466 | 23.4 | 23.4 | 0.000 |
| **BMI at recruitment (kg/m^2^)** |  |  |  |  |  |  |
| <18.5 | 0.5 | 0.7 | 0.067 | 0.6 | 0.6 | 0.000 |
| 18.5-24.9 | 34.7 | 25.7 | 0.777 | 27.2 | 27.2 | 0.000 |
| 25-29.9 | 43 | 41.8 | 0.855 | 42.5 | 42.5 | 0.000 |
| ≥30 | 21.7 | 31.8 | 0.482 | 29.7 | 29.7 | 0.000 |
| **Lifestyle score category** |  |  |  |  |  |  |
| Most healthy | 60.7 | 51.5 | 1.181 | 53.9 | 53.9 | 0.000 |
| Moderately healthy | 37.3 | 44.6 | 0.727 | 42.8 | 42.8 | 0.000 |
| Least healthy | 2 | 3.9 | 0.120 | 3.3 | 3.3 | 0.000 |
| **Cardiovascular disease at recruitment** |  |  |  |  |  | 0.000 |
| No | 73.9 | 55.5 | 1.499 | 59.5 | 59.5 | 0.000 |
| Yes | 26.1 | 44.5 | 0.505 | 40.5 | 40.5 | 0.000 |
| **Charlson comorbidity index** |  |  |  |  |  |  |
| 0 | 66.5 | 16.9 | 1.537 | 23.2 | 23.2 | 0.000 |
| 1 | 24.3 | 32.1 | 0.535 | 37.7 | 37.7 | 0.000 |
| ≥2 | 9.2 | 51 | 0.060 | 39.1 | 39.1 | 0.000 |

**Supplementary Table 8. Event rates and adjusted hazard ratio for all-cause and cause-specific premature mortality among participants without cancer at recruitment, comparing any sepsis to participants without sepsis**

| **Model** | **No sepsis (ref)** | **Any sepsis** |
| --- | --- | --- |
| **All-cause mortality** |  |  |
| Events/person‐years | 4612/4435885 | 3957/545127 |
| Model 1—Sepsis (adjusted for sociodemographic factors) | 1.00 (Ref) | 7.33 (7.01-7.66) |
| Model 2 (adjusted for sociodemographic factors and health status) | 1.00 (Ref) | 2.41 (2.29-2.53) |
| Model 3 (adjusted for sociodemographic factors, health status, and lifestyle score) | 1.00 (Ref) | 2.39 (2.28-2.51) |
| **Cardiovascular mortality** |  |  |
| Events/person‐years | 1962/4416820 | 1264/522342 |
| Model 1—Sepsis (adjusted for sociodemographic factors) | 1.00 (Ref) | 4.81 (4.47-5.18) |
| Model 2 (adjusted for sociodemographic factors and health status) | 1.00 (Ref) | 2.34 (2.15-2.54) |
| Model 3 (adjusted for sociodemographic factors, health status, and lifestyle score) | 1.00 (Ref) | 2.32 (2.14-2.52) |
| **Respiratory mortality** |  |  |
| Events/person‐years | 252/4404201 | 518/515502 |
| Model 1—Sepsis (adjusted for sociodemographic factors) | 1.00 (Ref) | 16.61 (14.19-19.45) |
| Model 2 (adjusted for sociodemographic factors and health status) | 1.00 (Ref) | 7.90 (6.63-9.41) |
| Model 3 (adjusted for sociodemographic factors, health status, and lifestyle score) | 1.00 (Ref) | 7.77 (6.52-9.26) |
| **Cancer-related mortality** |  |  |
| Events/person‐years | 1843/4415687 | 1747/526246 |
| Model 1—Sepsis (adjusted for sociodemographic factors) | 1.00 (Ref) | 9.04 (8.44-9.68) |
| Model 2 (adjusted for sociodemographic factors and health status) | 1.00 (Ref) | 1.76 (1.64-1.88) |
| Model 3 (adjusted for sociodemographic factors, health status, and lifestyle score) | 1.00 (Ref) | 1.76 (1.64-1.89) |
| **Infection-related mortality** |  |  |
| Events/person‐years | 51/4402700 | 160/512731 |
| Model 1—Sepsis (adjusted for sociodemographic factors) | 1.00 (Ref) | 29.21 (21.07-40.47) |
| Model 2 (adjusted for sociodemographic factors and health status) | 1.00 (Ref) | 10.04 (6.99-14.43) |
| Model 3 (adjusted for sociodemographic factors, health status, and lifestyle score) | 1.00 (Ref) | 9.85 (6.85-14.15) |
| **Other-cause mortality** |  |  |
| Events/person‐years | 504/4405853 | 268/513630 |
| Model 1—Sepsis (adjusted for sociodemographic factors) | 1.00 (Ref) | 4.86 (4.16-5.68) |
| Model 2 (adjusted for sociodemographic factors and health status) | 1.00 (Ref) | 4.37 (3.66-5.22) |
| Model 3 (adjusted for sociodemographic factors, health status, and lifestyle score) | 1.00 (Ref) | 4.32 (3.62-5.16) |

Model 1 was adjusted for age at baseline, gender, ethnicity, education, Townsend deprivation index, BMI at baseline.

Model 2 was adjusted for age at baseline, gender, ethnicity, education, Townsend deprivation index, BMI at baseline, CVD, diabetes mellitus, Charlson comorbidity index.

Model 3 was age at baseline, gender, ethnicity, education, Townsend deprivation index, BMI at baseline, CVD, diabetes mellitus, Charlson comorbidity index, lifestyle score.

**Supplementary Table 9. Adjusted hazard ratio for all-cause and cause-specific premature mortality, comparing any sepsis to participants without sepsis, using** **propensity score for sepsis**

| **Mortality type** | **No sepsis (ref)** | **Any sepsis** |
| --- | --- | --- |
| All-cause mortality | 1.00 (Ref) | 2.33 (2.19-2.47) |
| Cardiovascular mortality | 1.00 (Ref) | 2.38 (2.12-2.67) |
| Respiratory mortality | 1.00 (Ref) | 5.74 (4.51-7.31) |
| Cancer-related mortality | 1.00 (Ref) | 1.77 (1.63-1.93) |
| Infection-related mortality | 1.00 (Ref) | 7.93 (4.72-13.33) |
| Other-cause mortality | 1.00 (Ref) | 4.18 (3.12-5.59) |

Adjustment for age at baseline, gender, ethnicity, education, Townsend deprivation index, BMI at baseline, CVD, diabetes mellitus, Charlson comorbidity index, lifestyle score.

**Supplementary Table 10. Adjusted hazard ratio for all-cause and cause-specific premature mortality, comparing any sepsis to participants without sepsis, using multiple imputation**

| **Mortality type** | **No sepsis (ref)** | **Any sepsis** |
| --- | --- | --- |
| All-cause mortality | 1.00 (Ref) | 2.41 (2.30–2.51) |
| Cardiovascular mortality | 1.00 (Ref) | 2.42 (2.23–2.62) |
| Respiratory mortality | 1.00 (Ref) | 7.48 (6.39–8.76) |
| Cancer-related mortality | 1.00 (Ref) | 1.79 (1.68–1.90) |
| Infection-related mortality | 1.00 (Ref) | 9.92 (7.10–13.84) |
| Other-cause mortality | 1.00 (Ref) | 4.45 (3.73–5.29) |

Adjustment for age at baseline, gender, ethnicity, education, Townsend deprivation index, BMI at baseline, CVD, diabetes mellitus, Charlson comorbidity index, lifestyle score.

**Supplementary Table 11. Adjusted hazard ratio for cause-specific premature mortality, comparing any sepsis to participants without sepsis, using Fine–Gray** **subdistribution hazards model**

| **Mortality type** | **No sepsis (ref)** | **Any sepsis** |
| --- | --- | --- |
| Cardiovascular mortality | 1.00 (Ref) | 2.35 (2.17–2.55) |
| Respiratory mortality | 1.00 (Ref) | 7.13 (5.95–8.56) |
| Cancer-related mortality | 1.00 (Ref) | 1.72 (1.62–1.83) |
| Infection-related mortality | 1.00 (Ref) | 9.36 (6.44–13.59) |
| Other-cause mortality | 1.00 (Ref) | 4.22 (3.52–5.05) |

Adjustment for age at baseline, gender, ethnicity, education, Townsend deprivation index, BMI at baseline, CVD, diabetes mellitus, Charlson comorbidity index, lifestyle score.

**Supplementary Table 12. Adjusted hazard ratio for all-cause and cause-specific premature mortality, comparing any sepsis to participants without sepsis, stratified by key characteristics**

|  | **No sepsis (ref)** | **Any sepsis** | ***P* value for interaction** |
| --- | --- | --- | --- |
| **All-cause mortality** |  |  |  |
| Age |  |  | <0.001 |
| ≤54 | 1.00 (Ref) | 0.76 (0.62-0.92) |  |
| 55-59 | 1.00 (Ref) | 1.61 (1.48-1.75) |  |
| 60-64 | 1.00 (Ref) | 2.66 (2.46-2.88) |  |
| ≥65 | 1.00 (Ref) | 3.15 (2.94-3.37) |  |
| Charlson comorbidity index (CCI) |  |  | <0.001 |
| CCI=0 | 1.00 (Ref) | 1.94 (1.85-2.04) |  |
| CCI=1 | 1.00 (Ref) | 3.74 (3.22-4.34) |  |
| CCI≥2 | 1.00 (Ref) | 4.26 (3.91-4.64) |  |
| Lifestyle score category |  |  | <0.001 |
| Most healthy | 1.00 (Ref) | 2.04 (1.73-2.41) |  |
| Moderately healthy | 1.00 (Ref) | 2.33 (2.18-2.48) |  |
| Least healthy | 1.00 (Ref) | 2.45 (2.30-2.61) |  |
| **Cardiovascular mortality** |  |  |  |
| Age |  | 0.85 (0.61-1.19) | <0.001 |
| ≤54 | 1.00 (Ref) | 1.54 (1.32-1.80) |  |
| 55-59 | 1.00 (Ref) | 2.74 (2.39-3.15) |  |
| 60-64 | 1.00 (Ref) | 3.18 (2.82-3.59) |  |
| ≥65 | 1.00 (Ref) |  |  |
| Charlson comorbidity index (CCI) |  |  | 0.001 |
| CCI=0 | 1.00 (Ref) | 2.01 (1.57-2.57) |  |
| CCI=1 | 1.00 (Ref) | 2.21 (2.00-2.45) |  |
| CCI≥2 | 1.00 (Ref) | 2.98 (2.63-3.39) |  |
| Lifestyle score category |  |  | <0.001 |
| Most healthy | 1.00 (Ref) | 2.02 (1.55-2.63) |  |
| Moderately healthy | 1.00 (Ref) | 2.27 (2.03-2.52) |  |
| Least healthy | 1.00 (Ref) | 2.55 (2.26-2.87) |  |
| **Respiratory mortality** |  |  |  |
| Age |  | 2.33 (1.23-4.4) | <0.001 |
| ≤54 | 1.00 (Ref) | 4.56 (3.45-6.04) |  |
| 55-59 | 1.00 (Ref) | 8.90 (6.63-11.95) |  |
| 60-64 | 1.00 (Ref) | 10.75 (8.23-14.05) |  |
| ≥65 | 1.00 (Ref) |  |  |
| Charlson comorbidity index (CCI) |  |  | <0.001 |
| CCI=0 | 1.00 (Ref) | 3.92 (3.22-4.77) |  |
| CCI=1 | 1.00 (Ref) | 11.93 (9.20-15.47) |  |
| CCI≥2 | 1.00 (Ref) | 15.49 (10.75-22.31) |  |
| Lifestyle score category |  |  | 0.0913 |
| Most healthy | 1.00 (Ref) | 7.65 (5.93-9.88) |  |
| Moderately healthy | 1.00 (Ref) | 7.04 (5.66-8.77) |  |
| Least healthy | 1.00 (Ref) | 6.66 (4.06-10.92) |  |
| **Cancer-related mortality** |  |  |  |
| Age |  |  | <0.001 |
| ≤54 | 1.00 (Ref) | 0.58 (0.44-0.77) |  |
| 55-59 | 1.00 (Ref) | 1.20 (1.07-1.35) |  |
| 60-64 | 1.00 (Ref) | 1.88 (1.69-2.10) |  |
| ≥65 | 1.00 (Ref) | 3.16 (3.77-4.26) |  |
| Charlson comorbidity index (CCI) |  |  | <0.001 |
| CCI=0 | 1.00 (Ref) | 1.60 (1.51-1.70) |  |
| CCI=1 | 1.00 (Ref) | 4.18 (3.51-4.99) |  |
| CCI≥2 | 1.00 (Ref) | 4.67 (2.63-8.31) |  |
| Lifestyle score category |  |  | <0.001 |
| Most healthy | 1.00 (Ref) | 1.09 (0.84-1.42) |  |
| Moderately healthy | 1.00 (Ref) | 1.67 (1.53-1.82) |  |
| Least healthy | 1.00 (Ref) | 1.94 (1.79-2.11) |  |
| **Infection-related mortality** |  |  |  |
| Age |  |  | <0.001 |
| ≤54 | 1.00 (Ref) | 0.70 (0.21-2.28) |  |
| 55-59 | 1.00 (Ref) | 10.42 (5.64-19.25) |  |
| 60-64 | 1.00 (Ref) | 10.55 (5.26-21.17) |  |
| ≥65 | 1.00 (Ref) | 13.98 (8.03-24.34) |  |
| Charlson comorbidity index (CCI) |  |  | 0.011 |
| CCI=0 | 1.00 (Ref) | 6.56 (4.46-9.66) |  |
| CCI=1 | 1.00 (Ref) | 14.17 (5.98-33.57) |  |
| CCI≥2 | 1.00 (Ref) | 20.55 (10.18-41.46) |  |
| Lifestyle score category |  |  | 0.0683 |
| Most healthy | 1.00 (Ref) | 8.51 (5.47-13.22) |  |
| Moderately healthy | 1.00 (Ref) | 9.44 (2.67-33.37) |  |
| Least healthy | 1.00 (Ref) | 11.95 (6.84-20.87) |  |
| **Other-cause mortality** |  |  |  |
| Age |  |  | <0.001 |
| ≤54 | 1.00 (Ref) | 0.50 (0.14-1.82) |  |
| 55-59 | 1.00 (Ref) | 3.91 (2.65-5.76) |  |
| 60-64 | 1.00 (Ref) | 4.70 (3.73-5.91) |  |
| ≥65 | 1.00 (Ref) | 4.99 (3.60-6.91) |  |
| Charlson comorbidity index (CCI) |  |  | 0.003 |
| CCI=0 | 1.00 (Ref) | 3.27 (2.34-4.57) |  |
| CCI=1 | 1.00 (Ref) | 4.79 (3.65-6.30) |  |
| CCI≥2 | 1.00 (Ref) | 4.92 (3.83-6.33) |  |
| Lifestyle score category |  |  | 0.787 |
| Most healthy | 1.00 (Ref) | 4.05 (3.11-5.27) |  |
| Moderately healthy | 1.00 (Ref) | 4.54 (3.58-5.76) |  |
| Least healthy | 1.00 (Ref) | 4.30 (2.41-7.66) |  |

Adjustment for age at baseline, gender, ethnicity, education, Townsend deprivation index, BMI at baseline, CVD, diabetes mellitus, Charlson comorbidity index, lifestyle score.

**Supplementary Table 13. Baseline characteristics of participants included and excluded from the cohort study**

| **Characteristic** | Included | Excluded | *P* value |
| --- | --- | --- | --- |
| **Participants** | 371558 | 130672 |  |
| **Sociodemographic** |  |  |  |
| **Gender** |  |  | <0.001 |
| Female | 194329 (52.3) | 78869 (60.4) |  |
| Male | 177229 (47.7) | 51803 (39.6) |  |
| **Age at recruitment (years)** |  |  |  |
| **Age group** |  |  | <0.001 |
| ≤54 | 149703 (40.3) | 44311 (33.9) |  |
| 55-59 | 67408 (18.1) | 23367 (17.9) |  |
| 60-64 | 88334 (23.8) | 33101 (25.3) |  |
| ≥65 | 66113 (17.8) | 29893 (22.9) |  |
| **Ethnicity** |  |  | <0.001 |
| White | 354784 (95.5) | 117672 (90.1) |  |
| Non-white | 16774 (4.5) | 10225 (7.8) |  |
| Missing | 0 | 2775 (2.1) |  |
| **Education** |  |  | <0.001 |
| College or University degree | 47471 (12.8) | 9370 (7.2) |  |
| Others | 324087 (87.2) | 111180 (85.1) |  |
| Missing | 0 | 10122 (7.8) |  |
| **Townsend deprivation index quintile** |  |  | 0.994 |
| 1 (least deprived) | 74564 (20.1) | 26204 (20.1) |  |
| 2 | 73875 (19.9) | 25940 (19.9) |  |
| 3 | 74444 (20) | 26259 (20.1) |  |
| 4 | 74372 (20) | 26146 (20.0) |  |
| 5 (most deprived) | 74303 (20) | 26123 (20.0) |  |
| **BMI at recruitment (kg/m^2^)** |  |  | <0.001 |
| <18.5 | 1882 (0.5) | 743 (0.6) |  |
| 18.5-24.9 | 124820 (33.6) | 37473 (28.7) |  |
| 25-29.9 | 159325 (42.9) | 52681 (40.3) |  |
| ≥30 | 85531 (23) | 36672 (28.1) |  |
| Missing | 0 | 3103 (2.4) |  |
| **Lifestyle** |  |  |  |
| **Smoking status** |  |  | <0.001 |
| Never | 204460 (55) | 68909 (52.7) |  |
| Previous | 130324 (35.1) | 42639 (32.6) |  |
| Current | 36774 (9.9) | 16176 (12.4) |  |
| Missing | 0 | 2948 (2.3) |  |
| **Alcohol drinking** |  |  | <0.001 |
| Never | 13648 (3.7) | 8719 (6.7) |  |
| Previous | 12226 (3.3) | 5868 (4.5) |  |
| Current | 345684 (93) | 114433 (87.6) |  |
| Missing | 0 | 1652 (1.3) |  |
| **Health status** |  |  |  |
| **Cardiovascular disease at recruitment** |  |  | <0.001 |
| No | 266048 (71.6) | 84677 (64.8) |  |
| Yes | 105510 (28.4) | 43775 (33.5) |  |
| Missing | 0 | 2220 (1.7) |  |
| **Charlson comorbidity index** |  |  | <0.001 |
| 0 | 223611 (60.2) | 68685 (52.6) |  |
| 1 | 94036 (25.3) | 37265 (28.5) |  |
| ≥2 | 53911 (14.5) | 24722 (18.9) |  |

Data are presented as number (percentage), mean (SD), or median (P25, P75).

**
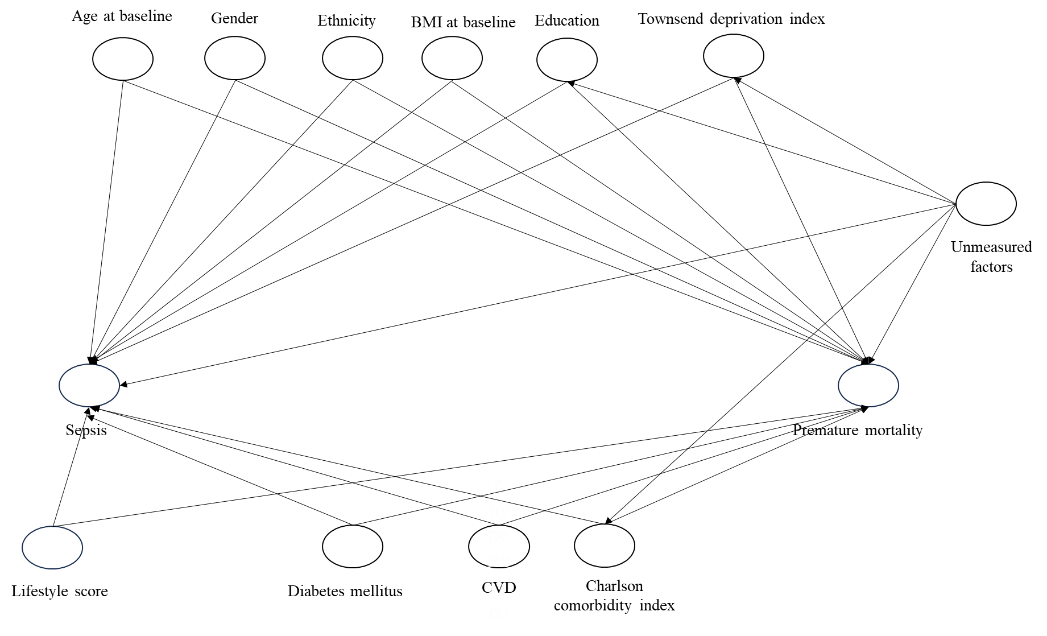
**

**Supplemental Figure 1. Directed Acyclic Graph depicting the hypothesized pathways between sepsis and the risk of premature mortality**
